# Electrochemical Degradation of Plastic Waste Coupled with Hydrogen Evolution in Seawater Using Rosette‐Like High‐Entropy Oxides

**DOI:** 10.1002/advs.202507023

**Published:** 2025-07-02

**Authors:** Zhenhao Xu, Yuchen Wang, Zhikeng Zheng, Xiaodie Zhang, Bin Liu, Karen Wilson, Kai Yan

**Affiliations:** ^1^ School of Environmental Science and Engineering Sun Yat‐sen University Guangzhou 510275 China; ^2^ School of Environment and Science Griffith University Gold Coast QLD 4222 Australia

**Keywords:** electrochemical, hydrogen evolution, OH* species, plastic degradation, seawater

## Abstract

Plastic overproduction and improper disposal generates over 390 million tons of waste annually, severely polluting marine ecosystems. Polyglycolic acid (PGA) is widely used in biomedical and packaging fields. Here, this study introduces an electrochemical degradation strategy for PGA waste that couples its conversion with seawater‐driven hydrogen evolution reaction (HER) using rosette‐like high‐entropy NiCoFeMnAlO_x_ nanosheets (r‐NCFMAO). The PGA‐derived glycolic acid oxidation reaction (GAOR) achieves 100 mA cm^−2^ at 1.36 V versus RHE, benefiting from abundant hydroxyl species (OH*) that lower the required potential by over 190 mV compared to the oxygen evolution reaction. This method produces ≈90% CO_3_^2−^, and subsequent Ca^2+^ precipitation recovers 77% of CaCO_3_, a valuable material in construction and papermaking. Electrochemical analysis, quasi in situ electron paramagnetic resonance, and in situ Raman spectroscopy reveal continuous OH^−^ oxidation enhancing GAOR activity, while density functional theory confirms that OH* lowers the energy barrier for the rate‐determining C─C bond cleavage and C─H bond activation. The integrated GAOR ‖ HER system sustains performance for over 300 h at industrial current densities and is applicable to upcycling various plastics. This work pioneers a synergistic approach for plastic waste valorization and hydrogen production, advancing circular carbon strategies.

## Graphical abstract

Rosette‐like high‐entropy NiCoFeMnAlO_x_ enable the electrochemical degradation of PGA plastic waste in seawater, coupling glycolic acid oxidation with hydrogen evolution. Abundant OH* species lower energy barriers for C─C and C─H bond activation, while in situ and theoretical studies reveal the catalytic mechanism. This work advances high‐entropy materials in sustainable plastic degradation and clean energy conversion.

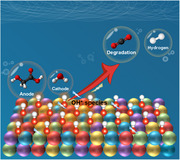

## 1 Introduction

Global plastic waste production was estimated at 390 million tons in 2021, with projections suggesting an alarming increase to 950 million tons annually by midcentury,^[^ ^1^, ^2^ ^]^ with over 8 million tons of plastic waste entering the oceans each year.^[^ ^3^, ^4^, ^5^ ^]^ In marine environments, polymer waste is nearly indestructible, disrupting carbon cycling processes and posing significant risks to ecological systems and human well‐being.^[^ ^6^ ^]^ While traditional thermal catalytic methods are effective for processing plastic waste collected on land, effective methods to degrade plastics in seawater are urgently sought.^[^ ^7^, ^8^ ^]^ Addressing this challenge necessitates the development of sustainable technologies for plastic degradation, with electrochemical methods gaining attention as a promising green approach, offering benefits such as operational simplicity, high efficiency, and environmental compatibility.^[^ ^9^ ^]^

Recently, an increasing number of promising studies have emerged on the electrochemical degradation of plastic waste,^[^ ^10^, ^11^, ^12^ ^]^ which involve pretreating plastics through chemical depolymerization followed by electrochemical oxidation of the organic monomer. Studies have shown that the inert C(sp^3^)─C/H(sp^3^) bonds can be electrocatalytically activated to promote upcycling of plastics,^[^ ^13^ ^]^ in which oxidation or reduction of plastic hydrolysates occurs, similar to the conventional electrocatalytic conversion of organic molecules.^[^ ^14^ ^]^ Generally, two main types of catalysts are used in electrocatalysis, noble metal and their based catalysts (e.g., Au, Pt, and Pd)^[^ ^15^, ^16^, ^17^ ^]^ and non‐noble metal catalysts (e.g., Ni, Co, and Fe).^[^ ^18^, ^19^ ^]^ Ethylene glycol, one of the monomers produced from polyethylene terephthalate (PET) depolymerization, can be highly selectively converted to the higher‐value glycolic acid (GA) at the Au/Ni(OH)_2_ electrode with 91% selectivity and 12 h durability.^[^ ^20^ ^]^ However, noble metal catalysts are limited by resource availability and are prone to poisoning during reactions,^[^ ^21^ ^]^ so developing non‐noble metal catalysts is of great importance. While Co containing 1D coordination polymer, have been designed to electrocatalytically upgrade PET into potassium diformate with ∼80% selectivity,^[^ ^22^ ^]^ the current density is only 10 mA cm^−2^, reflecting challenges of poor chemical instability and low catalytic efficiency faced by non‐noble metal catalysts.^[^ ^23^ ^]^ If direct conversion of microplastics in seawater is to be addressed, then additional challenges from the high concentration of chloride ions and associated severe corrosion of electrodes need to be overcome.^[^ ^24^, ^25^ ^]^ Therefore, there is an urgent need to develop non‐noble metal catalysts with exceptional activity and durability for plastic degradation in seawater.

High‐entropy engineering has gained significant attention as a promising strategy to enhance the performance of electrocatalysts for organic molecule conversion,^[^ ^26^, ^27^ ^]^ and is thus expected to improve the electrocatalytic efficiency of plastic degradation. By incorporating metal elements that can increase their valence states through electron‐deficient species, high‐entropy materials facilitate the generation of active sites conducive to O─O bond formation.^[^ ^28^, ^29^ ^]^ By integrating a variety of distinct elements, typically in equimolar proportions, within single‐phase electrocatalysts, the configurational entropy is elevated.^[^ ^30^, ^31^ ^]^ This enables a range of surface interaction combinations, significantly improving both activity and stability in targeted reactions, particularly for the electrocatalysis of organic molecules.^[^ ^32^, ^33^, ^34^ ^]^ For instance, it has been reported that PdAgSn/PtBi high‐entropy alloys show 4.8 and 4.9 times higher mass activity than commercial Pt/C catalysts in the electrocatalytic oxidation of methanol and ethanol.^[^ ^33^ ^]^ Despite their potential as promising catalysts in electrocatalytic alcohol oxidation, there have been few reported applications in plastic degradation, with the underlying mechanisms of complex plastic hydrolysate electrocatalysis still poorly understood.

**Scheme 1:**
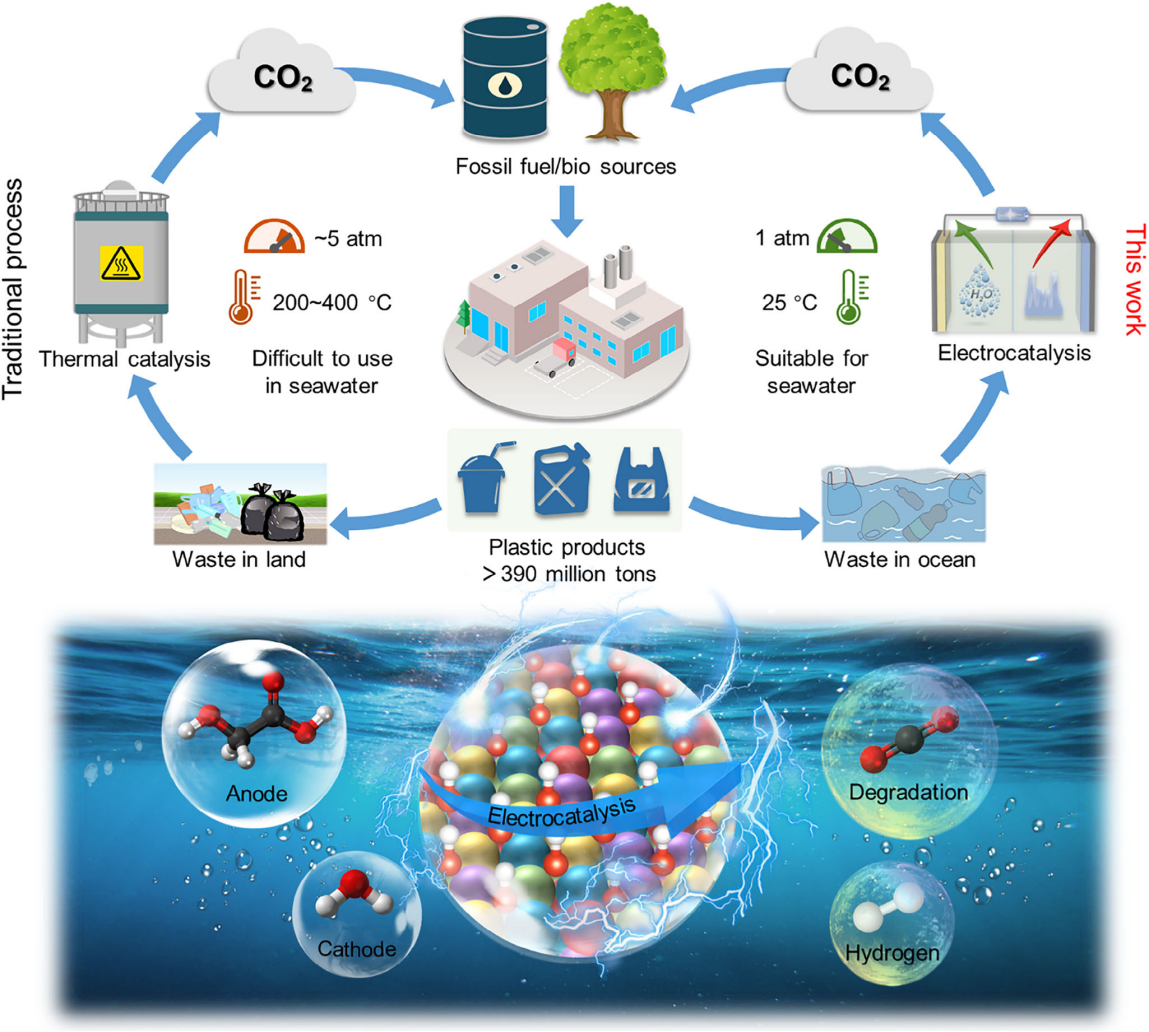
Carbon footprinting and electrocatalytic degradation of plastic waste in seawater. Background is modified from “Clear blue body of water” on Unsplash (https://unsplash.com/photos/XexawgzYOBc), free to use under the Unsplash License.

Herein, to address the gap, we have constructed the rosette‐like high‐entropy oxide NiCoFeMnAlO_x_ (r‐NCFMAO), through a facile hydrothermal method for high‐performance degradation of polyglycolic acid (PGA) plastic waste in seawater, facilitating the formation of a closed carbon cycle (**Scheme** **1**). By taking advantage of the abundant hydroxyl species (OH*) generated on r‐NCFMAO, the potential of glycolic acid oxidation reaction (GAOR) was significantly reduced by 190 mV at 100 mA cm^−2^ current density, compared to the oxygen evolution reaction (OER). At 1.4 V versus RHE potential, the current density of the high‐entropy r‐NCFMAO was >100 mA cm^−2^ higher than that of the non‐high‐entropy counterparts. PGA plastic waste was completely degraded and converted to CaCO_3_ with >77% yield, achieving a high performance that has not been previously reported. In situ spectroscopic studies (quasi in situ electron paramagnetic resonance (EPR), operando electrochemical impedance spectroscopy (EIS), and in situ Raman spectra) revealed the presence of high surface concentrations of active OH* species on r‐NCFMAO. A combination of experimental measurements and DFT calculations reveal these species facilitate C─C bond cleavage in glyoxalate and C─H bond oxidation during PGA degradation. In the paired system of GAOR and hydrogen evolution reaction (HER) in natural alkaline seawater, an impressive industrial‐level current density of 1.0 A cm^−2^ was achieved at a voltage of 1.96 V under harsh electrolysis conditions, with the system maintaining stable operation for over 300 h. The energy consumption was ≈3.8 kWh m^−3^ H_2_, which is lower than the typical range of 4.0–5.0 kWh m^−3^ H_2_ seen in conventional systems. This indicates a significant potential for practical applications. Furthermore, r‐NCFMAO demonstrated broad applicability for various plastic wastes, including PET, polybutylene terephthalate (PBT), polylactic acid (PLA), and polybutylene succinate (PBS), achieving over 80% degradation. This work presents a feasible strategy for the degradation of plastic waste in seawater.

## 2 Results and Discussion

### 2.1 Synthesis and Characterization

**Figure 1:**
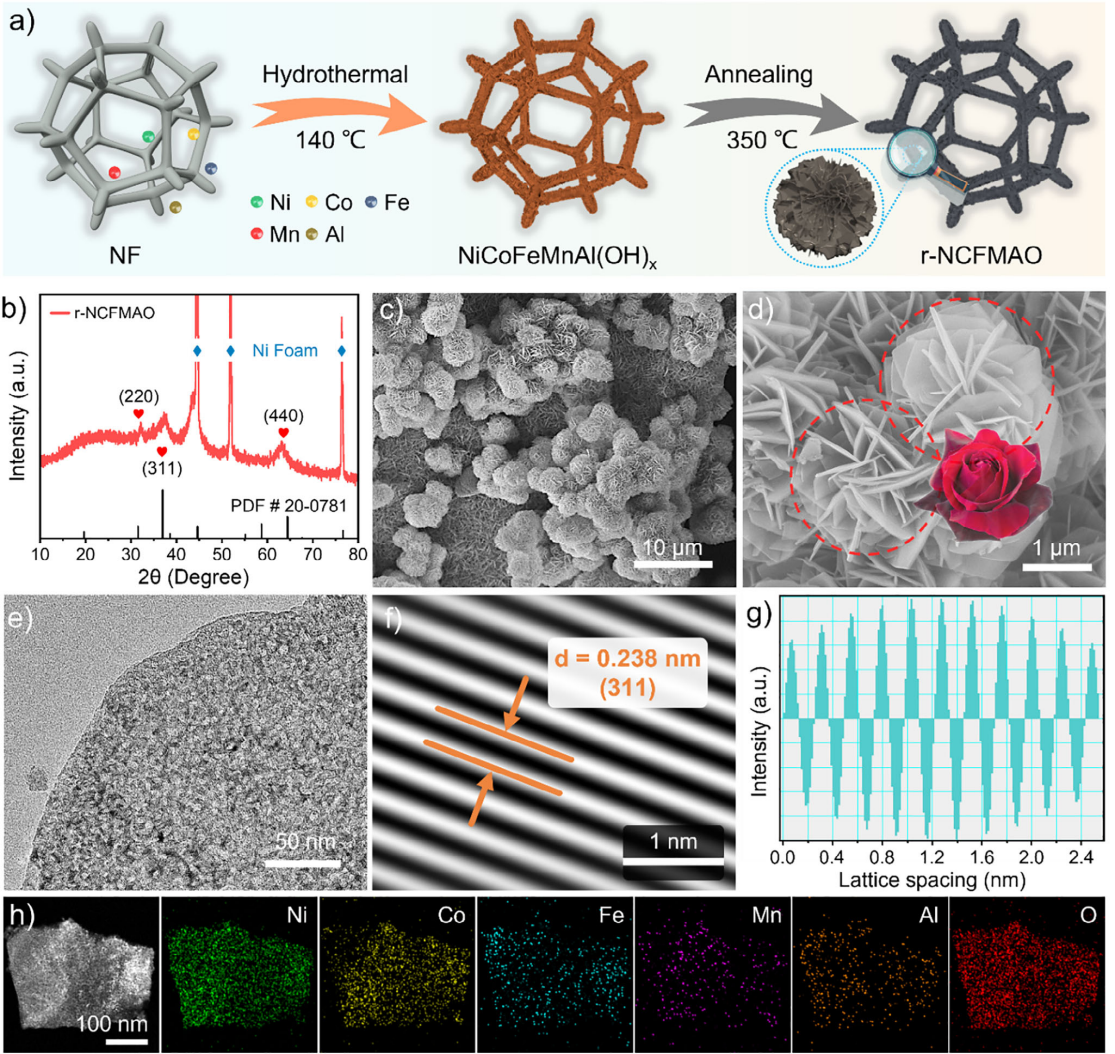
Synthesis and characterization of the r‐NCFMAO. a) Schematic illustration of the preparation. b) XRD patterns, c,d) SEM images, e) TEM image, f,g) lattice view through fast Fourier transforms from HRTEM image and h) EDS elemental mapping.

The r‐NCFMAO electrode was fabricated through the in situ growth of r‐NCFMAO on nickel foam (NF) using a facile hydrothermal method (**Figure** **1**a; Figure S1, Supporting Information). The X‐ray diffraction (XRD) pattern first showed the face‐centered cubic (FCC) spinel‐structured diffraction peaks (JCPDS#20‐0781) of the as‐prepared r‐NCFMAO, where peaks at 31.1°, 36.2°, and 64.1° could be attributed to the (220), (331), and (440) planes, respectively (Figure 1b). XRD patterns of the counterparts showed they were similar FCC structures (Figure S2, Supporting Information). The diffraction peak broadened when the elemental species increased, indicating that the grain size was getting smaller. The morphology and microstructure of the r‐NCFMAO were further characterized by scanning and transmission electron microscopy (SEM and TEM). In SEM images, the synthesized r‐NCFMAO particles were uniformly and compactly distributed on the surface of NF. Under enhanced magnification, the microscopic architecture exhibited distinctive rosette‐like nanosheets (Figure 1c,d; Figure S3, Supporting Information). The interlayer voids generated offer significant potential for enhancing substance diffusion and gaseous product evolution. TEM analysis was subsequently employed to elucidate the microstructural features, with images revealing the peripheral regions of the layer components exhibited numerous pores, which collectively contribute to an augmented specific surface area (Figure 1e). Lattice fringes could be observed in HRTEM images (Figure S4a, Supporting Information), for which fast Fourier transform produced lattice stripes with a crystal plane spacing of 0.238 nm, well matched to the (311) lattice plane of the spinel structure (Figure 1f,g). Selected area electron diffraction (SAED) analysis revealed distinct concentric diffraction patterns, unambiguously confirming the polycrystalline characteristics of r‐NCFMAO (Figure S4b, Supporting Information). Radial measurements of the diffraction rings established precise correspondence with the (220), (311), and (440) crystal planes of the spinel phase, consistent with prior XRD characterization data.^[^ ^35^ ^]^ Energy dispersive spectrometer (EDS) elemental mapping quantitatively verified the homogeneous spatial distribution of constituent elements, corroborating the chemical uniformity of the catalyst (Figure 1h). Inductively coupled plasma‐optical emission spectrometry (ICP‐OES) confirmed the atomic ratios of all metal elements, which showed close agreement to the cast ratios, which met the definition of a high‐entropy material (Figure S5, Supporting Information).^[^ ^36^ ^]^ X‐ray photoelectron spectroscopy (XPS) was then used to study the surface composition and chemical states of r‐NCFMAO (Figures S6 and S7, Supporting Information). The surface composition obtained by XPS was in general agreement with the ICP‐OES (Table S1, Supporting Information), in line with the previous report.^[^ ^37^ ^]^ Meanwhile, the metals were in the valence state of the corresponding oxides. Based on XPS analysis results, it could be concluded that the r‐NCFMAO should have strong electronic interaction, and the corresponding oxidation valence state existed to optimize the electric structure. Overall, the high‐entropy r‐NCFMAO assembled from porous nanosheets was successfully fabricated.

### 2.2 Performance of r‐NCFMAO

**Figure 2:**
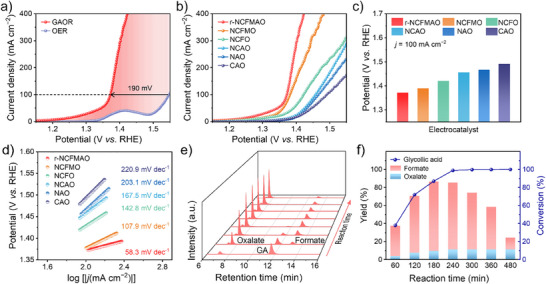
Electrochemical properties of GAOR. a) LSV curves of r‐NCFMAO in alkaline simulated seawater (1.0 M KOH + 0.5 M NaCl) with and without 0.1 M GA; b) Comparative LSV curves for the samples measured in alkaline simulated seawater with 0.1 M GA; c) The potentials and d) Tafel plots of different samples; e) HPLC chromatograms collected at various time points during a 480 min reaction; and f) GA conversion and the yields of formate and oxalate calculated from HPLC.

The GAOR performance of r‐NCFMAO was first evaluated in alkaline electrolytes in a standard three‐electrode H‐type cell. Linear sweep voltammetry (LSV) curves demonstrated that the current density increased sharply in the presence of 0.1 M GA at the same potential compared with OER in alkaline simulated seawater (**Figure** **2**a; Figure S8, Supporting Information). Specifically, the required applied potential to drive GAOR (1.36 V versus RHE) at a current density of 100 mA cm^−2^ was reduced by 190 mV in contrast to OER (1.55 V versus RHE), which can not only reduce energy consumption but also effectively avoid the occurrence of the chlorine oxidation reaction. Most importantly, the GAOR performance of r‐NCFMAO in alkaline simulated seawater was barely diminished compared to that in 1.0 M KOH (Figures S9–S11, Supporting Information). As shown in Figure 2b,c, the GAOR performance of r‐NCFMAO was superior to that of the quaternary oxide NiCoFeMnO_x_ (NCFMO) as well as significantly better than that of the ternary oxides and binary oxides. To verify the intrinsic catalytic performance, LSV measurements were also performed on carbon paper (CP) without iR compensation, with similar activity observed (Figure S12, Supporting Information). Subsequently, the Tafel slopes obtained by linear fitting of the polarization curves were used to evaluate the reaction kinetics of the GAOR process (Figure 2d). It was assumed that OH^−^ was not easily adsorbed on the catalyst to form OH* when the Tafel slope was above 120 mV dec^−1^, which was the rate‑determining step (RDS). When the slope was in the range of 40–120 mV dec^−1^, a smaller slope indicated a greater ability to generate OOH species.^[^ ^38^, ^39^ ^]^ The lower Tafel slope of r‐NCFMAO (58.3 mV dec^−1^) indicates enhanced electron transfer capability relative to NCFMO (107.9 mV dec^−1^) and NiCoFeO_x_ (NCFO, 142.8 mV dec^−1^), which is a consequence of the rapid generation of the active sites such as OH*. Furthermore, EIS measurements showed that r‐NCFMAO possessed the smallest charge transfer resistance (R_ct_) among all catalysts (Figure S13, Supporting Information), consistent with more rapid charge transfer and better conductivity. The electrochemically active area (ECSA) was obtained by the double‐layer capacitance (C_dl_) method with the help of cyclic voltammetry curves (CV) in non‐Faraday intervals (Figure S14, Supporting Information). Figure S15 (Supporting Information) shows that the C_dl_ of r‐NCFMAO (3.59 mF cm^−2^) was higher than those of NCFMO (1.77 mF cm^−2^) and NCFO (0.71 mF cm^−2^), demonstrating that it had larger ECSA to expose more active sites. The above results suggest that the r‐NCFMAO has potential for excellent electrocatalytic performance in GAOR and ultimate degradation of plastic waste in a seawater environment, which can be attributed to the abundant metal active sites and promotion of catalytic activity though the synergistic interactions between the multiple metal elements in the high‐entropy material surface.

The electrocatalytic properties of r‐NCFMAO were subsequently investigated by chronoamperometric tests, with the liquid products of GAOR analyzed qualitatively and quantitatively using high‐performance liquid chromatography (HPLC). Under three representative potentials, 1.40, 1.45, and 1.50 V versus RHE, GA was efficiently transformed to achieve an ≈100% conversion, with HPLC detecting formate as the main product alongside a small amount of oxalate (< 11% yield) (Figure 2e,f; Figures S16 and S17, Supporting Information). The highest formate Faraday efficiency of 45% was detected at a potential of 1.45 V versus RHE which corresponded to the highest formate yield (81%) production rate (0.79 mmol cm^−2^ h^−1^) in the first three hours of the reaction (Figure S18, Supporting Information). The low Faraday efficiency was attributed to the inevitable production of CO_3_^2−^ from the breaking of the GA C─C bond. It was noteworthy that as the electrolysis continued, the formate product underwent further conversion at 1.45 or 1.50 V versus RHE and was eventually completely consumed. While the oxalate by‐product did not degrade further, this is water‐soluble and can be safely managed or potentially utilized for value‐added applications. Separation of CO_3_^2−^ from the electrolyte by means of precipitation with calcium ions would make it possible to obtain the new materials CaCO_3_, which was widely used in plastics, building materials, paper making and other processes.

**Figure 3:**
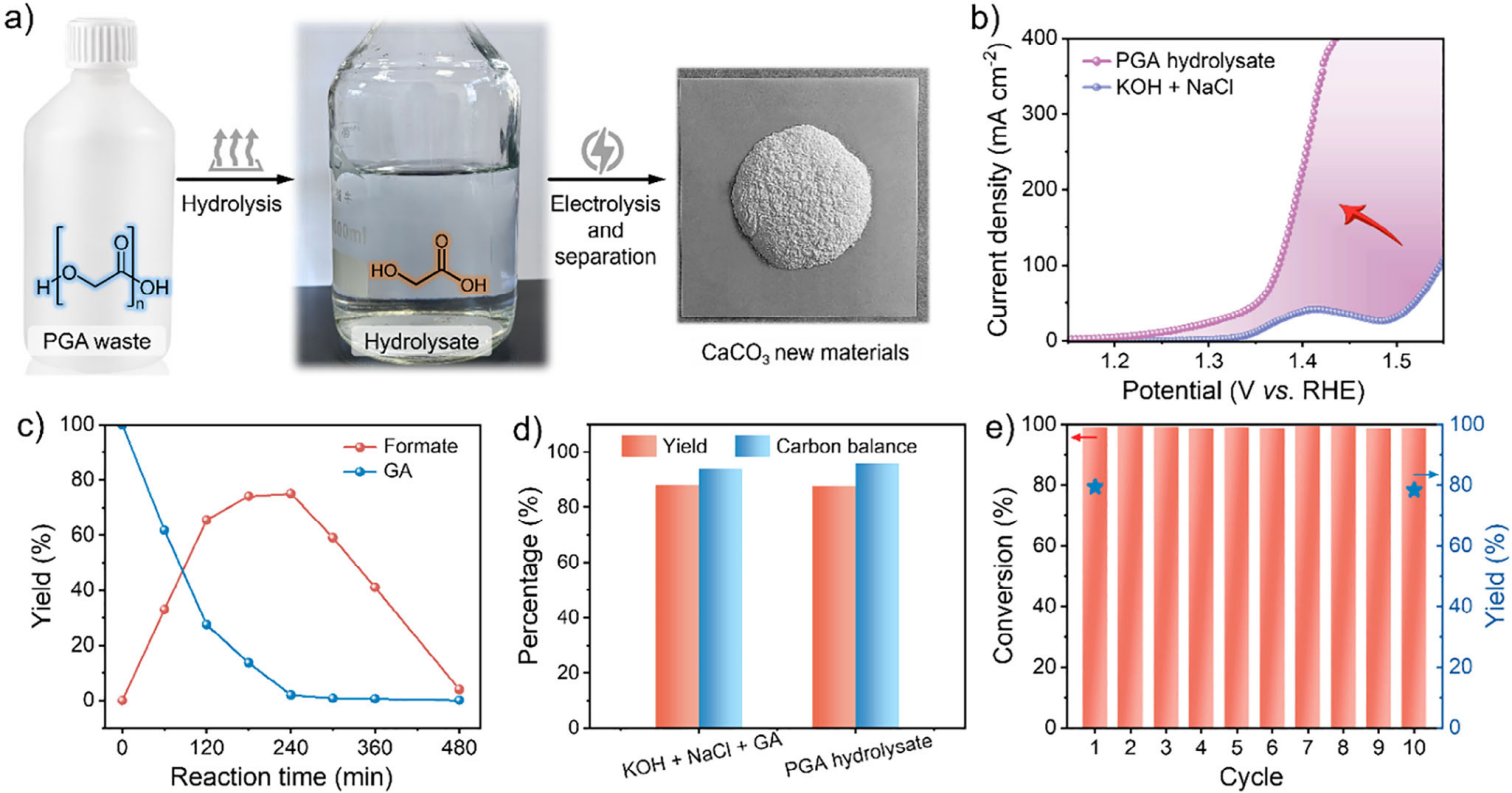
Distribution of electrocatalytic products from PGA hydrolysate. a) Diagram of the degradation of PGA waste; b) LSV curves of r‐NCFMAO in alkaline simulated seawater (1.0 M KOH + 0.5 M NaCl) with PGA hydrolysate; c) The substrate conversion and the product yield at 1.45 V versus RHE; d) Corresponding carbonate yield and carbon balance; and e) Recycle tests.

**Figure** **3**a shows a scheme for the overall process of taking PGA waste, depolymerizing the sample to form hydrolysate, followed by electrooxidation and then removal of CO_3_^2−^ by precipitation. Figure 3b shows PGA plastic waste was successfully hydrolyzed using alkaline simulated seawater as the electrolyte, and was quite striking that the electrochemical performance of the hydrolysate was almost comparable to that of using the GA pure substance. At the potential of 1.45 V versus RHE, the hydrolysate produced formate while maintaining a high conversion of GA, ≈100%, and later further conversion of formate also occurred (Figure 3c). The content of CO_3_^2−^ in solution was quantified by titration. The CO_3_^2−^ yield in the hydrolysate reached 90% with a Faraday efficiency of 85% and a production rate of 0.62 mmol cm^−2^ h^−1^, almost consistent with the use of GA‐pure substances (Figure 3d). The carbon balance exceeded 97%, representing an ≈3% improvement over values reported in prior studies.^[^ ^40^ ^]^ Upon addition of clarified Ca(OH)_2_ solution to the electrolyte, a large amount of white insoluble material appeared. After centrifugal separation and drying, the substance CaCO_3_ was detected by XRD and no other diffraction peaks were evident. The final recovery of the new material CaCO_3_ was 77%. Combining the product analysis results and the reported work, the total reaction pathway can be deduced as shown in Figure S19 (Supporting Information). To further elucidate the reaction pathway of GAOR, the electrochemical activity of key intermediates were evaluated using LSV measurements (Figure S20, Supporting Information). Glyoxylic acid, a representative oxidation intermediate derived from PGA, exhibited notable anodic current on the r‐NCFMAO catalyst, indicating its electrochemical reactivity. In contrast, oxalate, another detected species, showed negligible activity under the same conditions, suggesting that it is a terminal by‐product rather than an active intermediate. These results, together with the HPLC data showing no significant change in oxalate concentration during electrolysis, support the proposed degradation pathway. Comparing the potentials of other non‐precious metals used for plastic degradation at a current density of 10 mA cm^−2^ reveals this work shows excellent performance, even at potentials reduced by 200 mV (Figure S21, Supporting Information). To assess the longevity of the process the hydrolysate was then continuously refreshed for cyclic stability assessment. The r‐NCFMAO catalyst was operated continuously in the harsh alkaline electrolyte and maintained high activity over 10 cycles with refresh electrolyte (Figure 3e; Figure S22, Supporting Information), demonstrating the excellent stability of rosette‐like structure high‐entropy r‐NCFMAO catalysts. The electrochemical performance of GAOR after cycling was further tested, with LSV curves indicating minimal loss of performance. (Figure S23, Supporting Information). The r‐NCFMAO catalysts after GAOR maintained the FCC structure as revealed by XRD and preserved the rosette shape by SEM characterization (Figures S24 and S25, Supporting Information). Post‐reaction XPS analysis (Figure S26, Supporting Information) confirmed the stability of the surface chemical states, suggesting minimal reconstruction during electrolysis. The above results indicated that the r‐NCFMAO catalysts had excellent structural stability in GAOR. Thus we suggest the complex multi‐element composition of r‐NCFMAO imparts excellent structural stability to the catalyst, with the high‐entropy matrix enhancing corrosion resistance, preventing phase segregation during prolonged operation, which maintains catalytic performance.^[^ ^37^ ^]^ The excellent performance of PGA degradation once again proved that the high‐entropy oxides exposed more active sites, essential for degrading plastic waste.

### 2.3 Mechanistic Insights of GAOR

**Figure 4:**
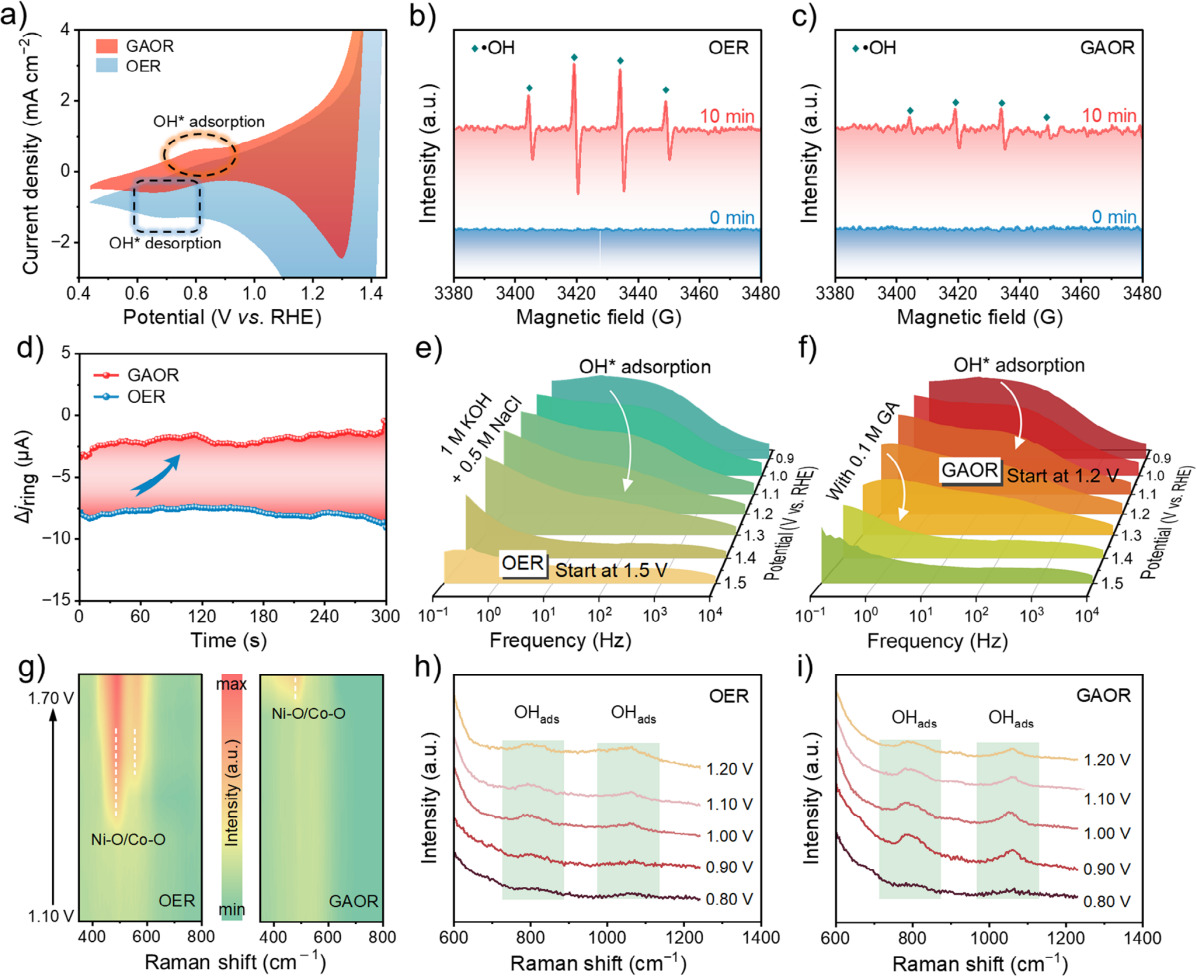
Investigations of GAOR mechanism on r‐NCFMAO catalyst. a) CV curves in alkaline simulated seawater with and without 0.1 M GA. EPR spectra of b) OER and c) GAOR using DMPO as a trapping agent. d) Ring current on a RRDE with a ring potential of 0.40 V in Ar‐saturated electrolyte without GA (blue) and with GA (red). Bode phase plots for in alkaline simulated seawater e) without and f) with 0.1 M GA. g) In situ Raman spectra during OER and GAOR at increased potentials. In situ Raman spectra during h) OER and i) GAOR in the wavenumber region 600∼1200 cm^−1^.

To understand the activity of GAOR, further tests using in situ measurements were performed. Based on previous studies,^[^ ^41^ ^]^ we speculated that the activity of the r‐NCFMAO for PGA conversion stemmed from the increased coverage of surface‐generated OH*, which was promoted by its special rosette‐like structure that increases the electrochemical surface area. The adsorption/activation of OH* species was first studied by CV measurements, (**Figure** **4**a) which shows an oxidation peak appears ≈0.85 V versus RHE in both GAOR and OER, corresponding to OH^−^ oxidation to adsorbed OH* species on the catalyst surface. The higher oxidation current of OH* in the GAOR process was more conducive to promoting the production of OH* than the OER process, resulting in higher OH* coverage and enhanced GAOR activity. The comparable GAOR activity in alkaline simulated seawater and 1.0 M KOH suggests that Cl^−^ has limited impact on OH* formation, and does not substantially compete with OH^−^ for active sites at 1.45 V versus RHE (which is below the potential for Cl^−^ oxidation). To identify OH* species on r‐NCFMAO, EPR experiments were carried out with the presence of 5,5‐dimethyl‐1‐pyrroline N‐oxide (DMPO) as spin trapping agents. A strong typical four‐line characteristic DMPO−·OH signal with an intensity ratio of 1:2:2:1 was detected both during OER and GAOR, implying the generation of ·OH evolution (Figure 4b,c). Notably, the number of ·OH detected in the GAOR process was significantly lower than in the OER process. Specifically, the concentration of ·OH detected after 10 min of constant potential reaction was 4.8 µM, compared to only 0.8 µM during GAOR. This suggested that ·OH was consumed at a faster rate during the GAOR process and that most of the OH* was used to participate in the degradation of PGA plastic derivatives, as proposed in related electrocatalytic studies.^[^ ^15^ ^]^

To further elaborate on the priority of oxidation, rotating ring‐disk electrode (RRDE) experiments were conducted. A potential was maintained on the ring and a current was applied to the disk to allow OER and GAOR to occur. As shown in Figure 4d, significantly lower on‐ring currents were detected during GAOR compared to OER, and thus the absence of oxygen reduction currents meant that GAOR occurs preferentially. To investigate interfacial behaviors, operando EIS experiments were conducted. In the absence of GA, the OER‐related interfacial charge transfer started at a potential > 1.40 V versus RHE (Figure 4e). When GA was added into the electrolyte, the Nyquist semicircle and the arched phase angle in the low‐frequency region appeared at lower potential of 1.20 V versus RHE (Figure 4f). This suggested the beginning of a Faradaic reaction, GAOR, which was consistent with the oxidation potential analyzed from the LSV curve. However, when further increasing the potential, the low‐frequency region appeared at 1.50 V versus RHE, indicating that OER occurred. Those operando EIS results suggested that the presence of PGA hydrolysate substantially reduced the peak phase angles at lower potentials, facilitating the interfacial charge transfer.

In situ Raman spectroscopy was also employed to monitor the r‐NCFMAO electrode surface during OER and GAOR and probe the structural evolution of the catalyst. Under OER conditions, the spectral baseline remained featureless below 1.40 V versus RHE, with characteristic vibrational signatures emerging only upon reaching this critical potential threshold (Figure 4g). Two distinct bands at 476 and 555 cm^−1^ progressively evolved with increasing polarization, corresponding to Ni–O and Co–O vibrations in the catalytically active NiOOH and CoOOH phases. Striking contrast was observed under GAOR operation, where these metal‐oxygen vibrations remained undetectable until >1.70 V versus RHE. This suggested that high valence Ni and Co species were reduced to low valence by GA, resulting in a delayed appearance of the high valence signal of NiOOH and CoOOH. Typically, FeOOH showed a vibrational signal at 670 cm^−1^, however, there was no corresponding signal detected during the reaction process, so FeOOH was not the main active species.^[^ ^42^, ^43^ ^]^ At the same time, signals related to the adsorbed hydroxyl species could be observed at 782 and 1050 cm^−1^ between 0.8 and 1.20 V versus RHE (Figure 4h,i), which were inherently weak due to low surface coverage and short lifetime.^[^ ^44^ ^]^ This further proved that r‐NCFMAO would continue to produce OH* after 0.85 V versus RHE. Moreover, during the GAOR process, the signal observed at 782 cm^−1^ was stronger than that of OER, which was consistent with the results of the electrochemical CV test. The enhanced OH*‐related coverage observed in GAOR compared to OER arises from the distinct reaction mechanisms and reactant environments involved in the two pathways. The surface coverage of OH* during OER is relatively low due to the conversion to O* and OOH* species taking place via a four‐electron pathway requiring higher potential.^[^ ^45^ ^]^ In contrast, during GAOR, OH* plays a more sustained and direct role in oxidizing organic intermediates, with literature suggesting OH* generated from water dissociation acts as an active oxidizing species derived at low potential from PGA hydrolysates.^[^ ^46^ ^]^ Thus, the organic environment facilitates the stabilization and involvement of OH* in multiple steps of the oxidation process, leading to stronger Raman signals and higher surface OH* coverage. Adsorbed Cl^−^ typically give rise to Raman bands at 250–275 cm^−1^.^[^ ^47^ ^]^ However, no such features were observed at 1.5 V versus RHE during either GAOR or OER (Figure S27, Supporting Information), indicating that Cl^−^ adsorption on the catalyst surface was effectively suppressed. The in situ Raman spectra thus indicated that the promotion of GAOR activity came from the generation of abundant OH*.

**Figure 5:**
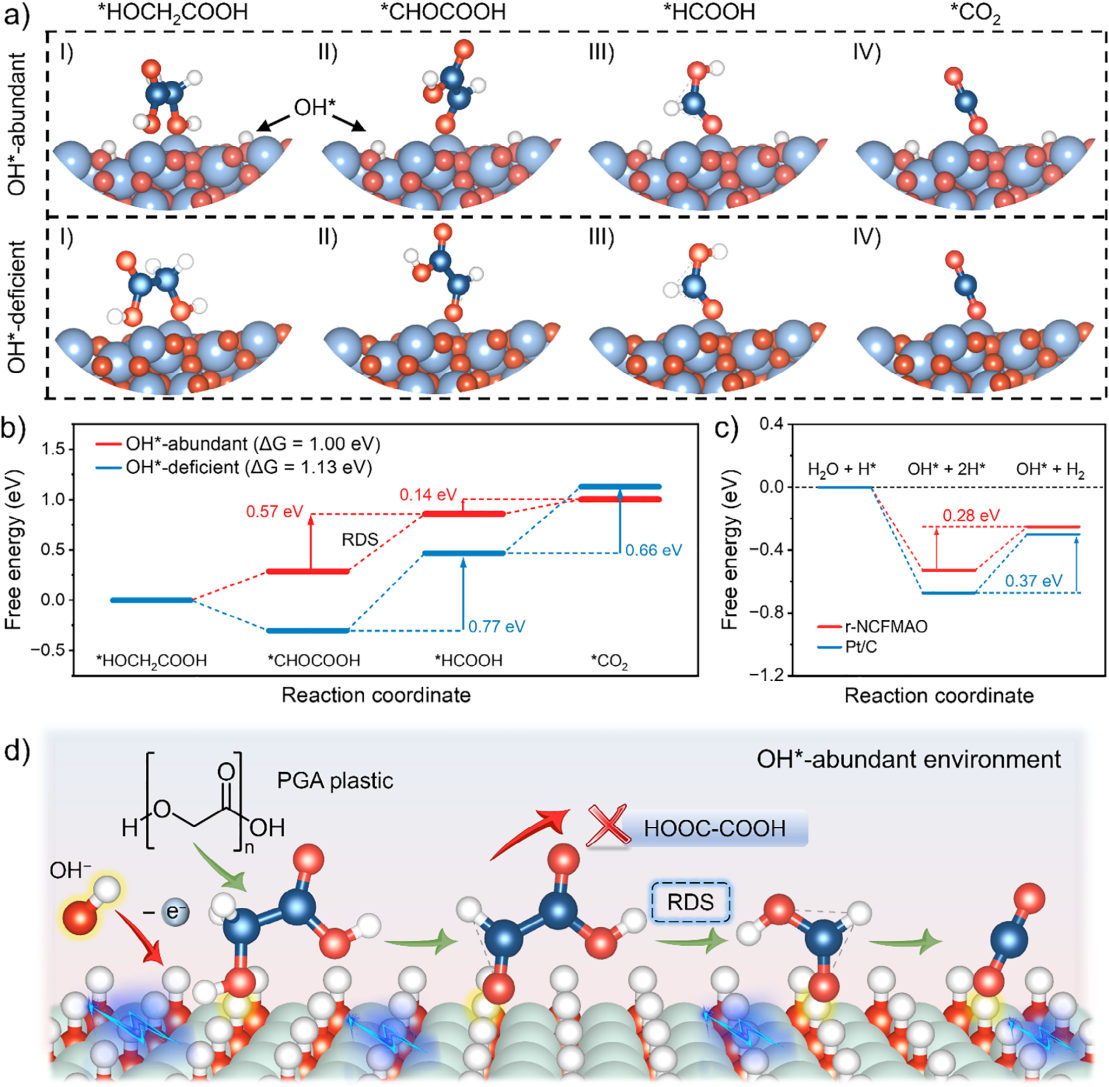
Theoretical calculations for the alkaline GAOR and HER. a) Calculated Gibbs free energies of *HOCH_2_COOH, *CHOCOOH, *HCOOH, and *CO_2_ intermediates; b) Corresponding free energy diagrams on the OH*‐deficient and OH*‐abundant surface of r‐NCFMAO; c) Free energy diagram of r‐NCFMAO and commercial Pt/C for the alkaline HER; and d) Schematic diagram of the conversion mechanism of PGA plastics by OH*‐rich r‐NCFMAO.

DFT calculations were then utilized to study the intrinsic mechanism. On the one hand, two models, OH*‐abundant and OH*‐deficient r‐NCFMAO surfaces, were constructed to illustrate the significance of the adsorbed OH* active species during GAOR (**Figure** **5**a). Based on the reaction pathways described above, the Gibbs free energies of the GAOR steps involving *HOCH_2_COOH, *CHOCOOH, *HCOOH, and *CO_2_ were calculated and formed into the step diagram shown in Figure 5b. The process in the overall reaction pathway that crossed the maximum energy barrier was the conversion of glyoxylate to formate, which involved the most difficult step of C─C bond cleavage. Conversion of formate to CO_2_, had a lower energy barrier over OH* rich surfaces, indicating OH* could effectively promote C─C bond cleavage and the activation of C─H bond of formate. This further demonstrates the active source of OH* is responsible for promoting the conversion of PGA plastics.

The potential of the r‐NCFMAO catalyst required for HER was also identified through theoretical calculations. As shown in Figure 5c, the Gibbs free energies of alkaline HER using r‐NCFMAO and commercial Pt/C catalysts were compared, which both needed to overcome the energy barrier for conversion of 2H* to H_2_, which was the RDS.^[^ ^48^, ^49^ ^]^ A smaller energy barrier of 0.28 eV was observed for r‐NCFMAO in this process, which was lower than that of the commercial Pt/C catalyst whose energy barrier was 0.37 eV, illustrating that r‐NCFMAO should show excellent performance in alkaline HER. This was experimentally tested, and as shown in Figure S28 (Supporting Information), the alkaline HER performance of r‐NCFMAO was comparable to that of commercial Pt/C. Thus, theoretical calculations support the critical role of OH* species in plastic waste degradation over r‐NCFMAO, with a suggested a mechanism for OH*‐rich r‐NCFMAO promoting the conversion of PGA plastics illustrated in Figure 5d.

### 2.4 Integrated Seawater Electrolyzer

**Figure 6:**
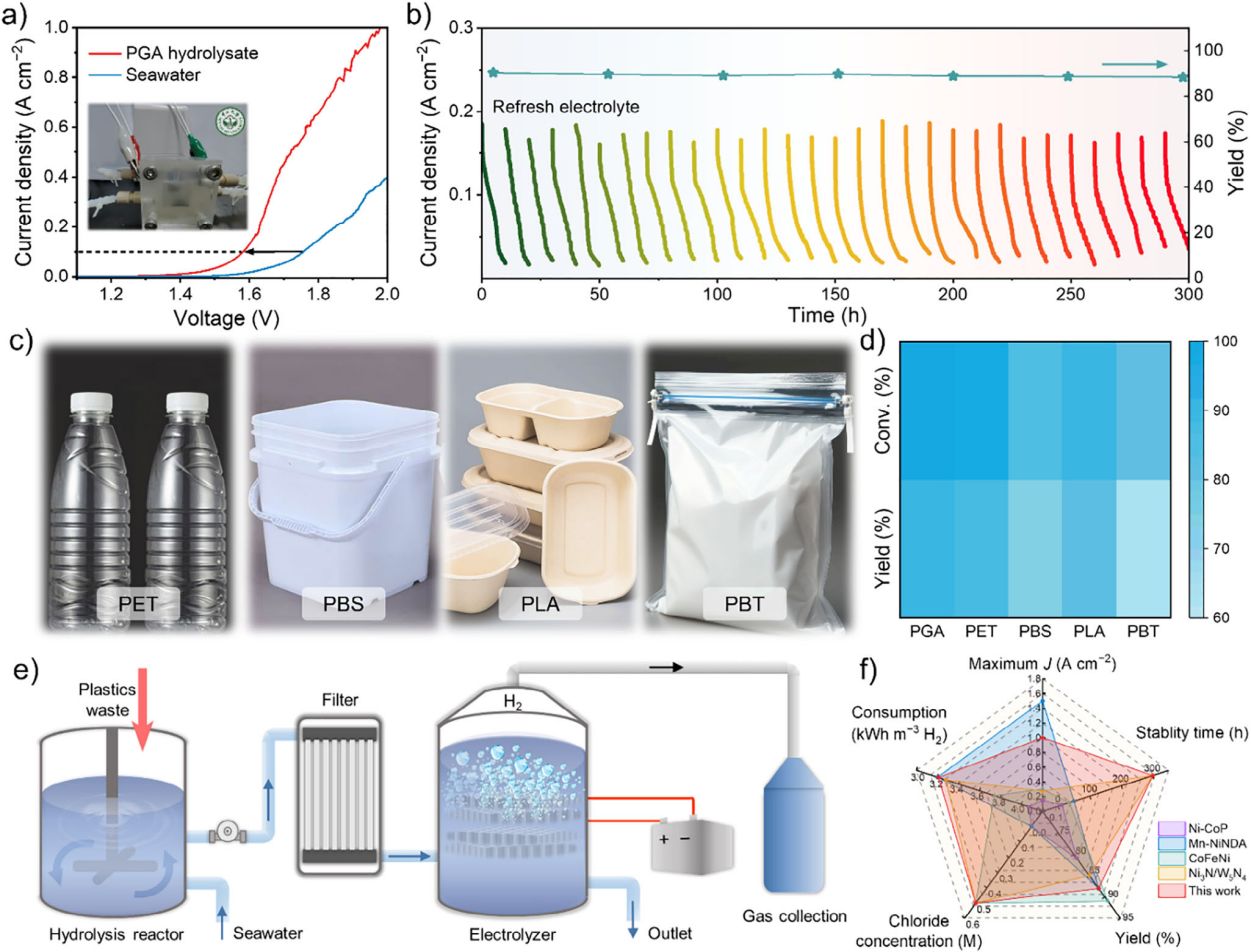
Simulation of industrial treatment of plastic waste and hydrogen production. a) LSV curves in natural alkaline seawater with and without PGA hydrolysate at 50 °C (Inset shows the electrolytic cell device); b) Chronoamperometric tests at 1.60 V under periodic electrolyte (natural alkaline seawater with PGA hydrolysate) renewal; c) Range of plastics (PET, PBS, PLA, and PBT) electrocatalytically degraded over r‐NCFMAO; d) Conversion and yield of a variety of plastics (PGA, PET, PBS, PLA and PBT); e) Schematic diagram of the operational flow for plastic degradation paired with hydrogen evolution; and f) Presentation of data on the strengths of this work.

The outstanding GAOR and HER properties make r‐NCFMAO a promising catalyst for the paired system. To further evaluate its performance, a flow cell electrolyzer was used to simulate industrial conditions, and its operational efficiency was investigated under the harsh conditions of an alkaline seawater environment at 50 °C.^[^ ^50^, ^51^ ^]^ This procedure used r‐NCFMAO as the anode for plastic degradation and as the cathode for hydrogen evolution (Figure S29, Supporting Information). As shown in **Figure** **6**a, the current density of the GAOR || HER pair could reach an industrial level of 1.0 A cm^−2^ at a low cell voltage of 1.96 V, significantly outperforming the OER || HER system. HPLC analysis of the anode product revealed a distribution pattern consistent with the standard three‐electrode system (Figure S31, Supporting Information). The energy consumption for production, evaluated from the LSV curves and measured H_2_ volume, demonstrated the electricity consumption of GAOR || HER was lower than the 5 kWh m^−3^ H_2_ observed for electrolytic (OER || HER) hydrogen production (Figures S31‐S33, Supporting Information). The energy consumption of the GAOR || HER system was calculated to be ≈3.80 kWh m^−3^ H_2_, which is lower than that of the conventional OER || HER system, measured under identical conditions to consume 4.31 kWh m^−3^ H_2_. Additionally, the energy required for PGA hydrolysis was reported to be ≈10% of the electricity consumption during electrolysis,^[^ ^52^ ^]^ demonstrating that integrating PGA oxidation with HER not only achieved plastic degradation but also reduced energy input compared to traditional water electrolysis. Surprisingly, the r‐NCFMAO catalyst remained highly stable over 300 h of continuous operation (Figure 6b). The conversion of plastic (100%), the yield of CO_3_^2−^ (90%), and the carbon balance (95%) remained consistent throughout the cycle, without any noticeable decrease. The versatility of r‐NCFMAO in degrading plastic hydrolysate in seawater was further investigated (Figure 6c,d; Figure S34, Supporting Information). The conversion of PET, PBS, PLA, and PBT was 99%, 91%, 82%, and 70% respectively, with the corresponding yields of 88%(CO_3_^2−^), 72%(succinate), 60%(acetate), and 35% (succinate) respectively observed. These plastic degradation processes were realized in the simulated process roadmap shown in Figure 6e. Challenges related to catalyst scale‐up, mass transport limitations, and long‐term system stability need to be addressed for future industrial implementation. In comparison to recently reported catalysts, r‐NCFMAO was superior in terms of both high current density and stability (Figure 6f),^[^ ^53^, ^54^, ^55^, ^56^ ^]^ suggesting r‐NCFMAO catalyst has potential for the large‐scale application in plastic degradation from seawater.

## 3 Conclusion

In summary, the rosette‐like high‐entropy oxide r‐NCFMAO, assembled from nanosheets, was designed for the simultaneous degradation of plastic waste and hydrogen generation in seawater. In situ electrochemistry test, EPR and in situ Raman demonstrated the generation of OH* active sites on r‐NCFMAO. The combination of theoretical calculations DFT and experimental results showed that the OH*‐rich r‐NCFMAO could effectively activate the C─C and C─H bonds of plastic intermediates, significantly enhancing the conversion of plastic into CO_3_^2−^. Simultaneously, its HER performance was comparable to that of the commercial Pt/C catalyst, resulting in a high current density of 1.0 A cm^−2^ at a low voltage of 1.96 V in a paired system. The catalytic durability of r‐NCFMAO for GAOR || HER remained over 300 h under harsh industrial conditions, exceeding the performance of other relevant reports by more than 100 h in seawater electrocatalysis. The energy consumption was ≈3.8 kWh m^−3^ H_2_, which was lower than the current conventional energy consumption (4.0–5.0 kWh m^−3^ H_2_), and therefore, has a great potential for practical applications. Compared with conventional catalysts, the superior performance of r‐NCFMAO can be attributed to its unique high‐entropy structure, which optimizes active site distribution and enhances electron transfer kinetics. This work is the first to report high‐entropy catalysts for realizing the plastic carbon cycle in seawater while simultaneously enabling energy‐saving hydrogen evolution, offering new insights into sustainable plastic degradation. Future research could focus on further optimizing the composition of high‐entropy materials and exploring their broader applicability in environmental catalysis and clean energy systems.

## Conflict of Interest

The authors declare no conflict of interest.

## Supporting information



Supporting Information

## Data Availability

The data that support the findings of this study are available from the corresponding author upon reasonable request.
