# Supplementary material for: Electrochemical Degradation of Plastic Waste Coupled with Hydrogen Evolution in Seawater Using Rosette‐Like High‐Entropy Oxides
Source: Adv Sci (Weinh). 2025 Jul 2;12(35):e07023. doi: 10.1002/advs.202507023 (PMC12463042; doi:10.1002/advs.202507023)
Supplement: Supplementary file 1 — Supporting Information [file ADVS-12-e07023-s001.docx]

Supporting Information

Electrochemical degradation of plastic waste coupling with hydrogen evolution in seawater using rosette-like high-entropy oxides

Zhenhao Xu, Yuchen Wang, Zhikeng Zheng, Xiaodie Zhang, Bin Liu, Karen Wilson,*
and Kai Yan*

Z. Xu, Dr. Y. Wang, Z. Zheng, X. Zhang, B. Liu, K. Yan
School of Environmental Science and Engineering, Sun Yat-sen University, Guangzhou 510275, China

E-mail: yank9@mail.sysu.edu.cn

K. Wilson
School of Environment and Science, Griffith University, Gold Coast QLD, Australia

E-mail: k.wilson6@griffith.edu.au

S1. Experimental Section

1.1 Chemicals and Materials

Nickel foam (NF, thickness of 1.0 mm) was obtained from Guangdong Canrd New Energy Technology Co., Ltd. Nickel nitrate hexahydrate (Ni(NO_3_)_2_·6H_2_O, 98%), manganese nitrate tetrahydrate (Mn(NO_3_)_2_·4H_2_O, 98%), aluminum nitrate nonahydrate (Al(NO_3_)_3_·9H_2_O, 99.99%), urea (CO(NH_2_)_2_, 99%), ammonium fluoride (NH_4_F, 98%), glycolic acid (C_2_H_4_O_3_, 70% aqueous solution) and polyglycolic acid (PGA) were obtained from Shanghai Aladdin Biochemical Technology Co., Ltd. Iron nitrate nonahydrate (Fe(NO_3_)_3_·9H_2_O, 99.99%), cobalt nitrate hexahydrate (Co(NO_3_)_2_·6H_2_O, 99.99%), potassium hydroxide (KOH, 95%), formic acid (CH_2_O_2_, 99%) and oxalic acid dihydrate (C_2_H_2_O_4_·2H_2_O, 99.5%) were obtained from Shanghai Macklin Biochemical Technology Co., Ltd. The commercial Pt/C catalyst (10 wt.%, with VXC72R conductive carbon black as carbon support) was obtained from Shanghai Hesen Electric Co., Ltd. Acetone (C_3_H_6_O, 99.5%), Hydrochloric acid (HCl, 36%) and sulfuric acid (H_2_SO_4_, 95%) were obtained from Guangzhou Chemical Reagent Factory. Absolute ethanol (C_2_H_6_O, 99.7%) was obtained from Guangdong Guanghua Sci-Tech Co., Ltd. Sodium chloride (NaCl, 99.5%) was obtained from Chengdu Chron Chemicals Co., Ltd. Natural seawater was obtained from the sea around Qingdao.

**1.2 Synthesis of r-NCFMAO**

The r-NCFMAO was grown on NF via a hydrothermal process and an annealing treatment step. Initially, a piece of NF (1.0 cm × 1.0 cm) was ultrasonically cleaned with acetone, 3.0 M hydrochloric acid, ultrapure water and ethanol in sequence. Fe(NO_3_)_3_·9H_2_O (0.30 mmol), Co(NO_3_)_2_·6H_2_O (0.60 mmol), Ni(NO_3_)_2_·6H_2_O (0.60 mmol), Mn(NO_3_)_2_·4H_2_O (0.75 mmol), Al(NO_3_)_3_·9H_2_O (0.30 mmol), NH_4_F (5.0 mmol) and urea (10.0 mmol) were dissolved in 30 mL ultrapure water to form a pink solution. Then, the prepared NF together with the above solution was transferred into a 50 mL autoclave and placed in an oven to raise the temperature to 140 ℃ and held for 16 h. Following the hydrothermal treatment, the NF with precursor was rinsed with ethanol for several times and dried in an oven at 60 ℃ for 4 h. After that, the precursor was calcined at 350 ℃ for 2 h in a muffle furnace in an air atmosphere to obtain r-NCFMAO. The other catalysts were prepared in the same way by deleting the metal salts of the corresponding casts.

**1.3 Structural Characterization**

X-ray diffraction (XRD) pattern was collected via a Rigaku Ultima IV X-ray diffractometer with Cu Kα radiation (40 kV and 40 mA) under a scan rate of 5° min^−1^. Scanning electron microscopy (SEM) images were obtained using a TESCAN MIRA LMS scanning electron microscope at 15 keV. Transmission electron microscopy (TEM) was conducted on an FEI Talos F200x microscope. X-ray photoelectron spectroscopy (XPS) measurements were recorded on a Thermo Scientific K-Alpha spectrometer equipped with an Al Kα radiation. Inductively Coupled Plasma Optical Emission Spectrometer (ICP-OES) was performed on Optima 5300 DV.

**1.4 Electrochemical Measurements**

All electrochemical measurements were carried out on a CHI 760E electrochemical workstation (Shanghai Chenhua Instrument Inc., China) at room temperature. The r-NCFMAO/NF was applied as the working electrode (1.0 cm^2^), platinum wire as the counter electrode and Hg|HgO (1.0 M KOH, aqueous) as the reference electrode, respectively. All potentials in this study were converted to the reversible hydrogen electrode (RHE) reference scale using the Nernst equation listed as follows: E (*vs.* RHE) = E (*vs.* Hg|HgO) + 0.098 + 0.059 × pH. Linear sweep voltametric (LSV) curves were determined in the corresponding electrolyte at a scan rate of 5 mV s^−1^ with 60% iR-compensation. Electrochemical impedance spectroscopy (EIS) measurements were performed over a frequency range from 100000 to 0.01 Hz with a perturbation of 5 mV. The electrochemical active surface area (ECSA) was estimated from the electric double-layer capacitance (C_dl_) obtained by cyclic voltammetry (CV) curves in a non-Faradic portion at various scan rates of 10, 20, 40, 60, 80 and 100 mV s^−1^. The value of ECSA was determined via the relationship: ECSA = S × C_dl_ / C_s_. In this expression, S corresponds to the geometric area of the working electrode, C_dl_ denotes the experimentally measured double-layer capacitance, and C_s_ represents the reference specific capacitance with an value of 0.04 mF cm^−2^. The electrochemical degradation process of GA or PGA hydrolysate were driven by chronoamperometry (CA) at the fixed potential of 1.45 V. The kinetic advantage between GAOR and OER was conducted on a rotating ring-disk electrode (RRDE) system. 4.0 mg r-NCFMAO powder was evenly distributed in 1000 μL mixed solution (960 μL ethanol and 40 μL 5 wt.% Nafion solution) and 10 μL of the ink was dropped on the RRDE (AF01WV10, Pine Research Instrumentation, USA). The potential of the ring electrode was set at 0.4 V to reduce newly-formed O_2_ during the electrooxidation reaction on the disk electrode and the current of the ring electrode was collected under the condition of Ar-saturated and 1600 rpm. In the flow cell test, two r-NCFMAO electrodes were used as anode and cathode. During the stability test, the electrolyte (natural alkaline seawater with PGA hydrolysate) was periodically renewed to replenish the depleted PGA.

**1.5 Hydrolysis of Plastic**

Plastic waste 0.5 g was immersed in 30 mL alkaline simulated seawater (1.0 M KOH + 0.5 M NaCl) or alkaline seawater (1.0 M KOH + seawater) and heated to 100 °C for 12 h with continuous stirring.

**1.6 Product Analysis**

The concentration of substrate and electrolytic products were analyzed quantitatively by high-performance liquid chromatography (HPLC, Shimadzu Prominence SIL-20A). The separation was performed on a Sepax Carbomix H-NP column at 55 ℃ using a 220 nm UV detector with a 2.5 mM H_2_SO_4_ mobile phase at a flow rate of 0.6 mL min^−1^. The concentration of CO_3_^2−^ was determined by acid-base titration. A 50 mL water sample was treated with 3 drops of phenolphthalein indicator, which turned pink in the presence of excess hydroxide ions. The sample was titrated with a 0.05 M HCl solution until the pink color disappeared, indicating the endpoint. The concentration of CO_3_^2−^ was calculated based on the volume of HCl consumed using the formula: c (CO_3_^2−^) = c (HCl) × V (HCl) / V (sample). The conversion and yield were calculated with the following equations.

Conversion (%) = (mole of consumed reactant/mole of initial reactant) × 100

Yield (%) = (mole of certain formed product/mole of initial reactant) × 100

**1.7 *In-Situ* Experiments**

EPR measurements were conducted using an EMX Plus spectrometer (Bruker, USA). The instrument settings included a center field of 3340 G, a sweep width of 200 G, and a sweep time of 10 seconds. The microwave power was adjusted to 65 mW, with a modulation amplitude of 2 G, receiver gain of 30 dB, and modulation frequency of 100 kHz. Data acquisition was performed with 1000 points. At the beginning (0 min), 50.0 µL of DMPO was introduced into the solution, which was then transferred to a capillary tube for EPR analysis. Additional samples were collected, and EPR measurements were repeated at subsequent time intervals.

Raman spectroscopy measurements were conducted using a LabRAM HR Evol. The r-NCFMAO electrode was used as the working electrode in 1.0 M KOH + 0.5 M NaCl with and without 0.1 M GA. The laser power was set to 50 mW. The catalyst was maintained at each potential for 300 seconds to allow the system to reach a steady state before the spectrum was recorded.

**1.8 Computational Methods**

All the calculations are performed in the framework of the density functional theory with the projector augmented plane-wave method, as implemented in the Vienna ab initio simulation package^[1]^. The generalized gradient approximation proposed by Perdew, Burke, and Ernzerhof is selected for the exchange-correlation potential^[2]^. The long-range van der Waals interaction is described by the DFT-D3 approach^[3]^. The cut-off energy for the plane wave is set to 500 eV. The energy criterion is set to 10^−6^ eV in the iterative solution of the Kohn-Sham equation. The effective U values of 5.3 eV, 3.3 eV, 6.2 eV, and 3.9 eV were applied to the d orbitals of Fe, Co, Ni, and Mn HEO to introduce the on-site Coulomb repulsion between d states in DFT+U approach. A vacuum layer of 15 Å is added perpendicular to the sheet to avoid artificial interaction between periodic images. The K-mesh resolved in real space is 0.04 2π/Å. All the structures are relaxed until the residual forces on the atoms have declined to less than 0.03 eV/Å.

**S2. Supporting Figures and Tables**


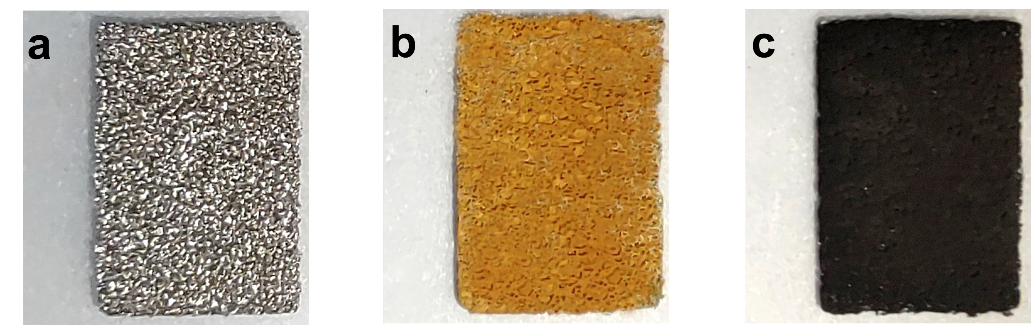


**Figure S1.** Digital images of a) NF, b) NiCoFeMnAl(OH)_x_/NF and c) r-NCFMAO/NF.


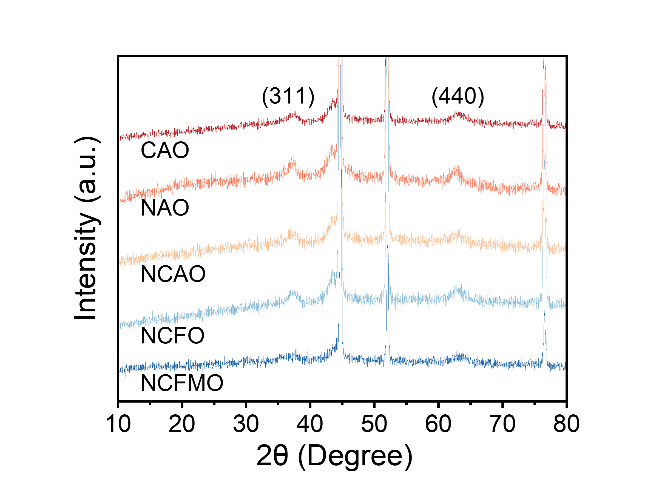


**Figure S2.** XRD patterns of different catalysts.

**
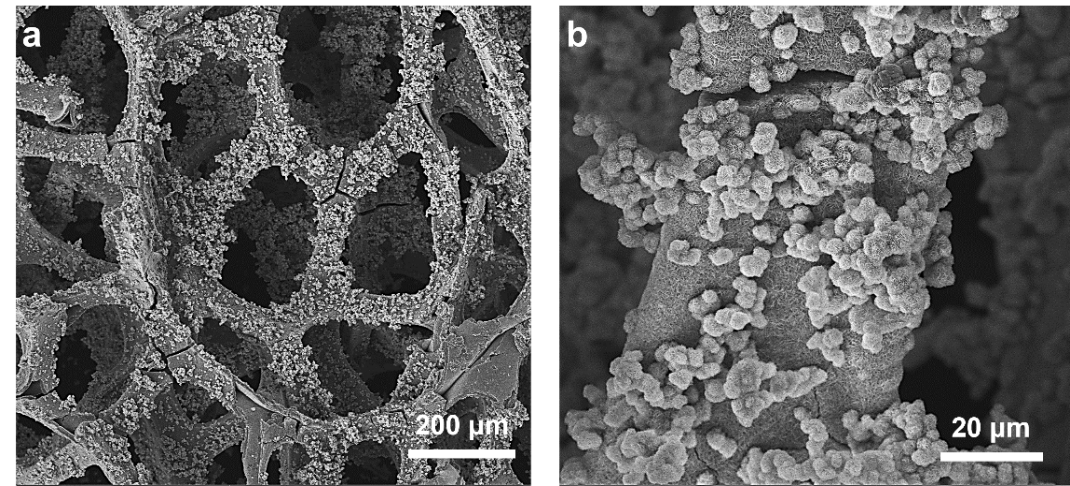
**

**Figure S3.** SEM images of r-NCFMAO/NF at different magnifications.

**
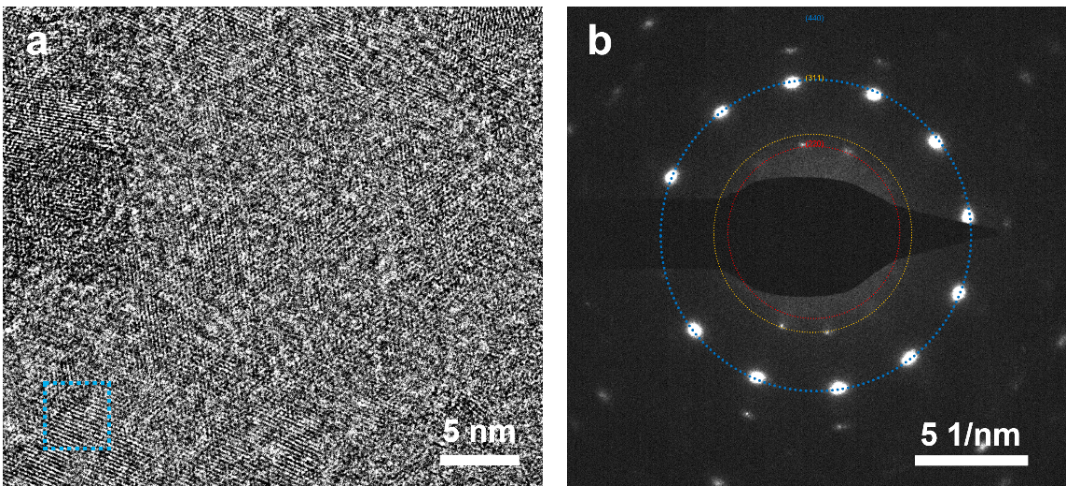
**

**Figure S4.** a) HRTEM image and b) SAED image of r-NCFMAO.

**
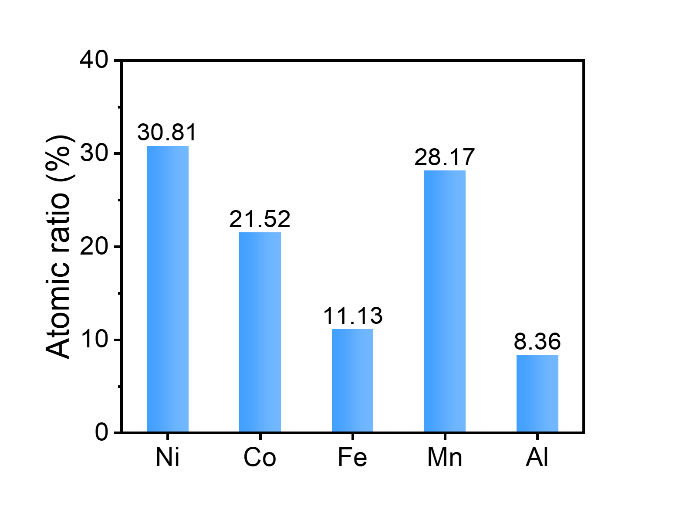
**

**Figure S5.** The atomic ratios of metallic elements in r-NCFMAO obtained from ICP-OES.

**
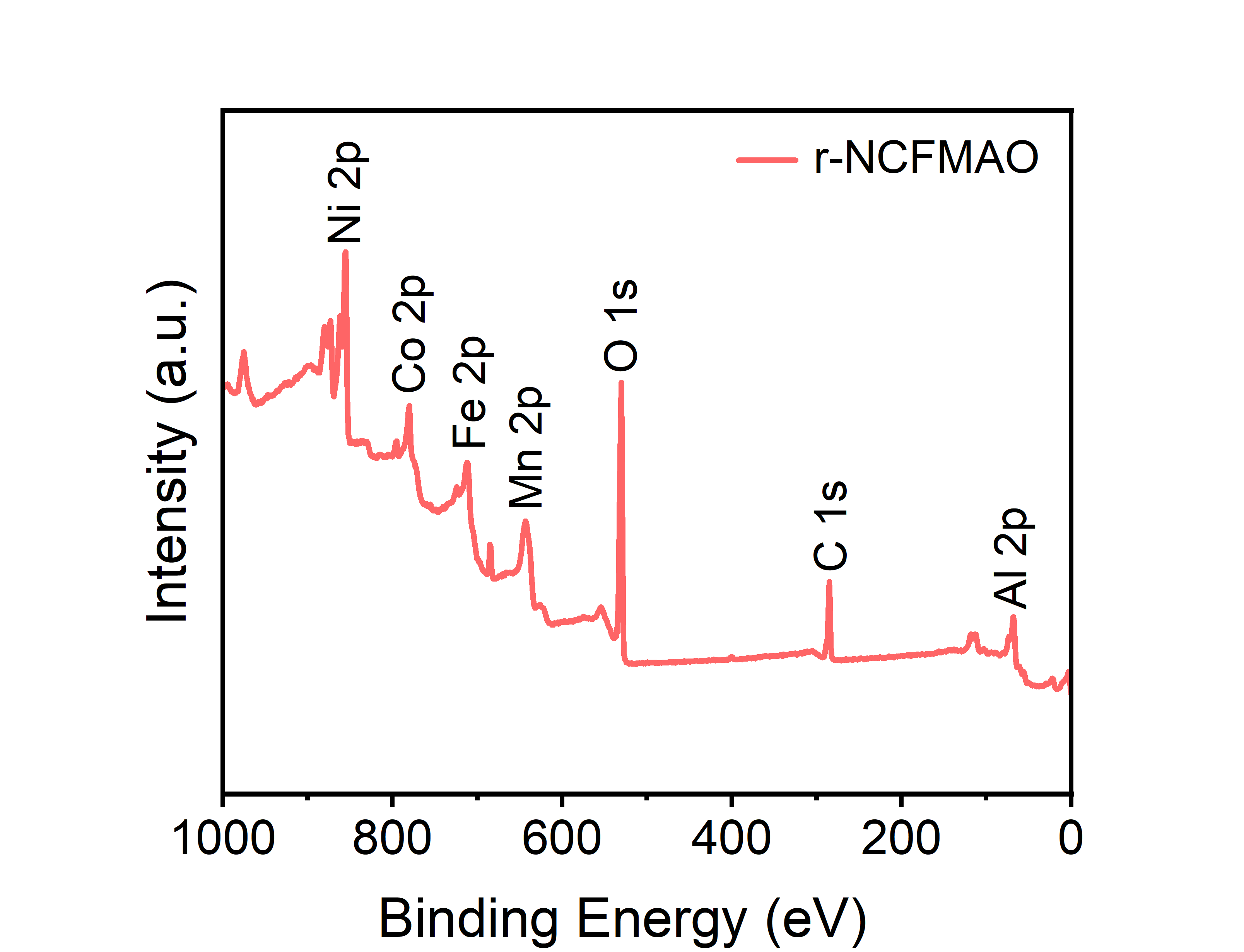
**

**Figure S6.** XPS survey spectra of r-NCFMAO


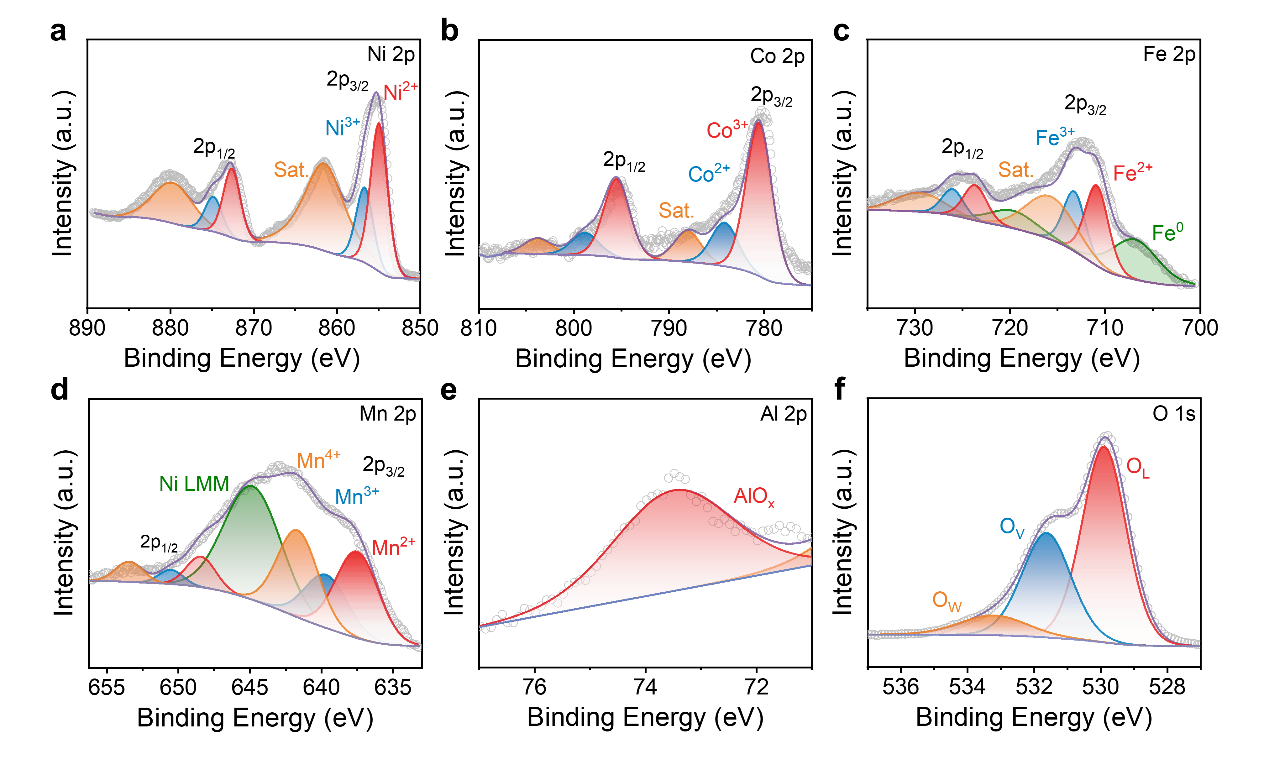


**Figure S7.** XPS spectra of r-NCFMAO.

**
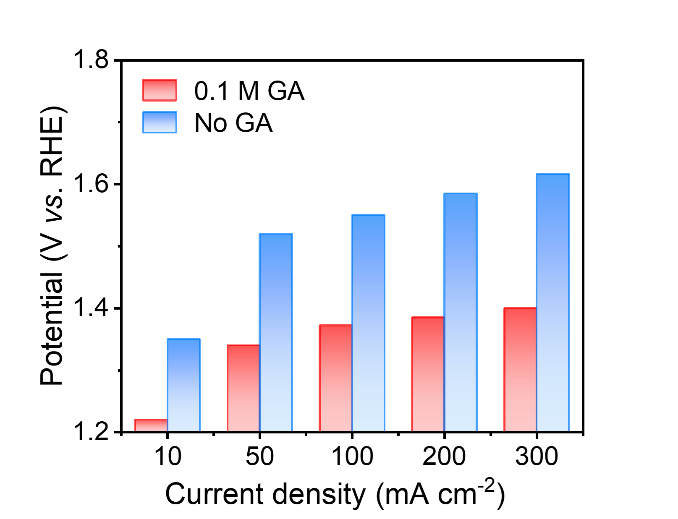
**

**Figure S8.** Comparison of potentials required to achieve the specified current densities of r-NCFMAO in alkaline simulated seawater (1.0 M KOH + 0.5 M NaCl) with and without 0.1 M GA.

**
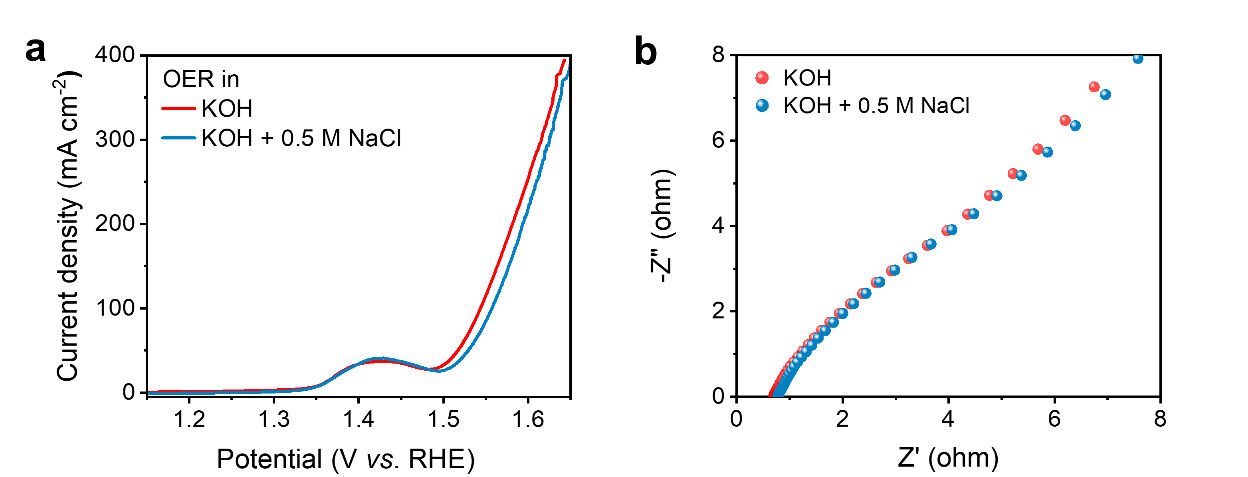
**

**Figure S9.** a) LSV curves and b) EIS plots of r-NCFMAO in KOH and alkaline simulated seawater.

**
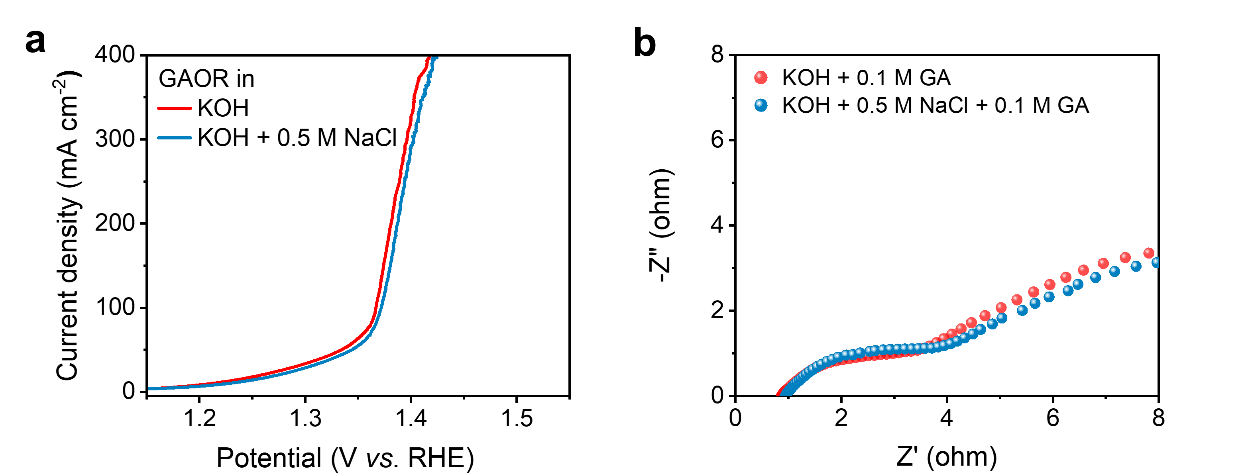
**

**Figure S10.** a) LSV curves and b) EIS plots of r-NCFMAO in KOH with 0.1 M GA and alkaline simulated seawater with 0.1 M GA.

**
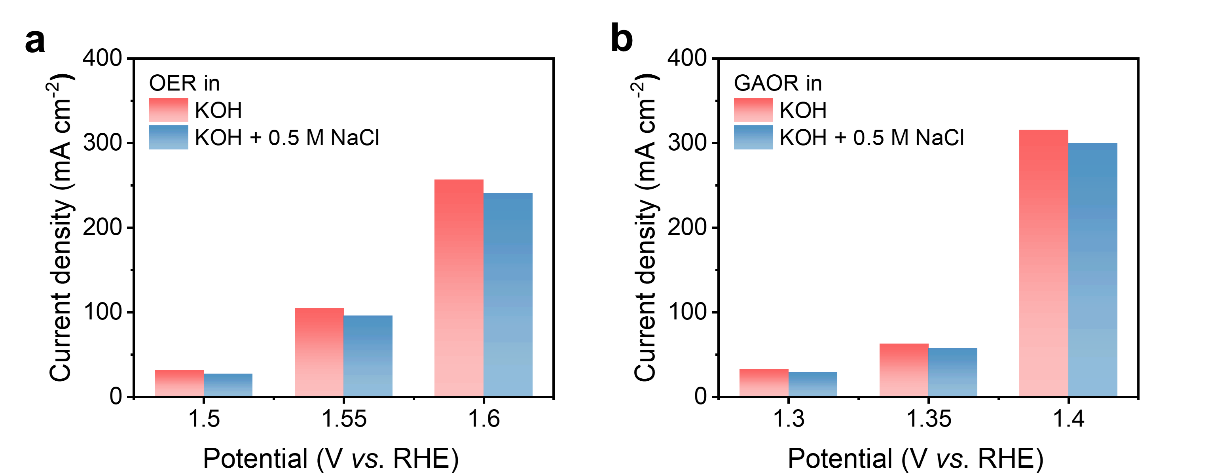
**

**Figure S11.** The current density of r-NCFMAO at different potentials for a) OER and b) GAOR.


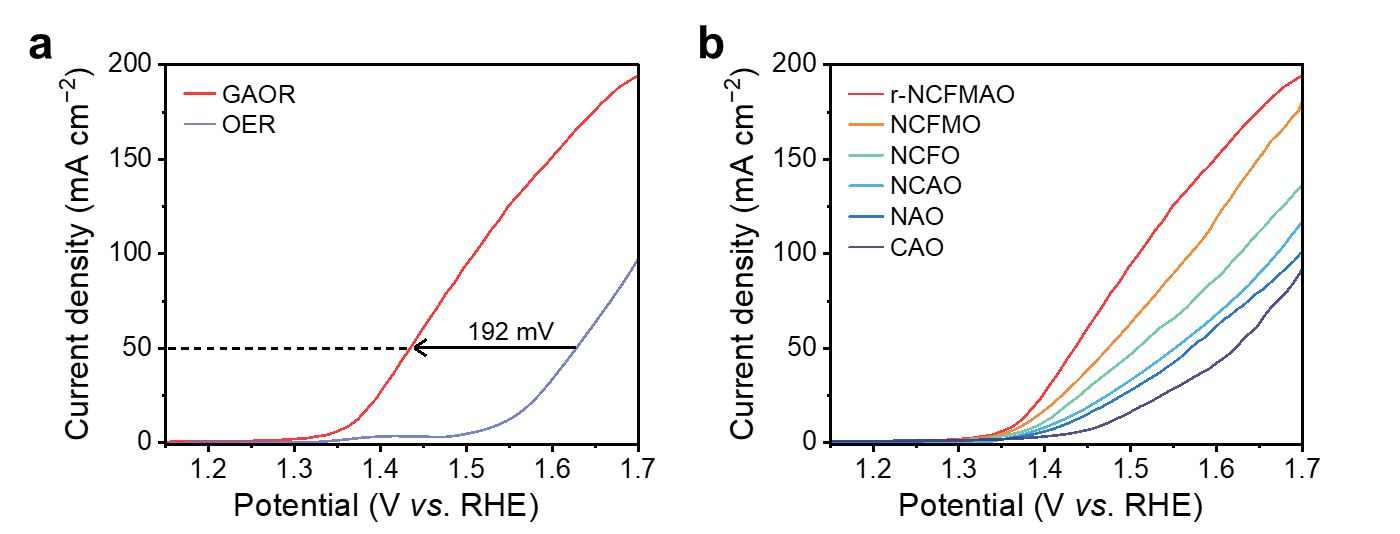


**Figure S12.** a) LSV curves of r-NCFMAO in alkaline simulated seawater (1.0 M KOH + 0.5 M NaCl) with and without 0.1 M GA. b) Comparative LSV curves for the samples measured in alkaline simulated seawater with 0.1 M GA. All catalysts were loaded on carbon paper, and the curves were without iR-compensation.


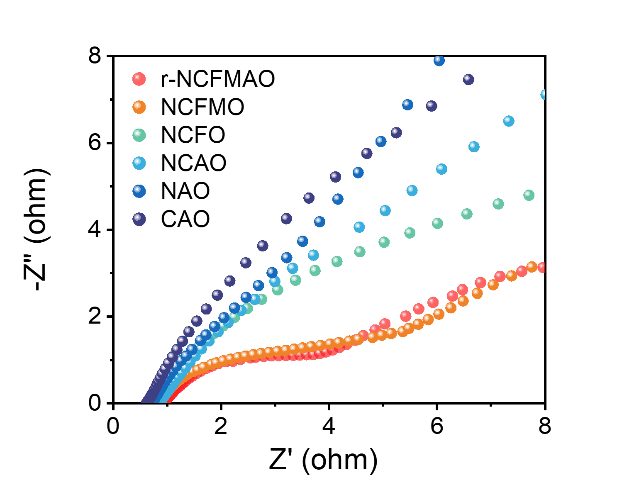


**Figure S13.** EIS tests of different catalysts in alkaline simulated seawater electrolyte (1.0 M KOH + 0.5 M NaCl + 0.1 M GA).

**
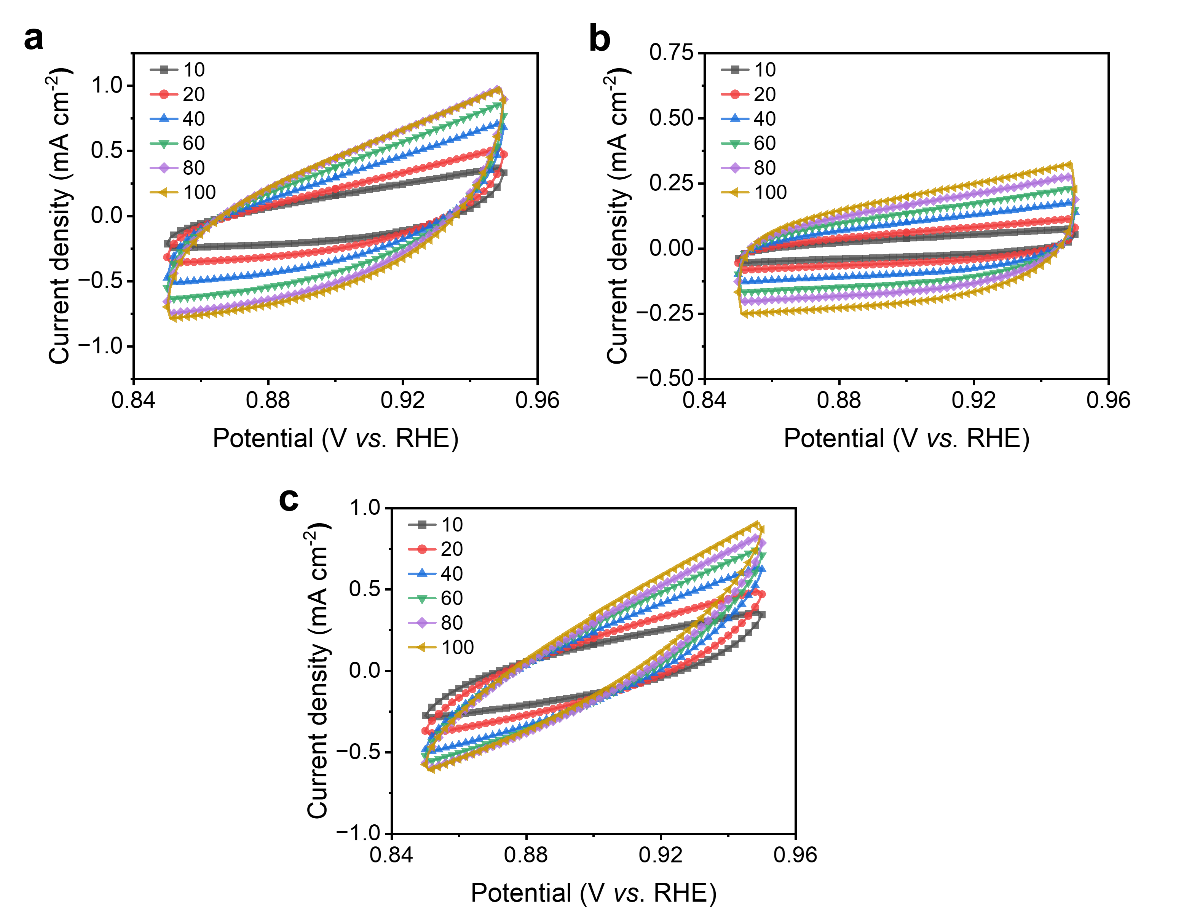
**

**Figure S14.** CV curves of a) r-NCFMAO, b) NCFMO and (c) NCFO in alkaline simulated seawater electrolyte (1.0 M KOH + 0.5 M NaCl + 0.1 M GA) at different scan rates.


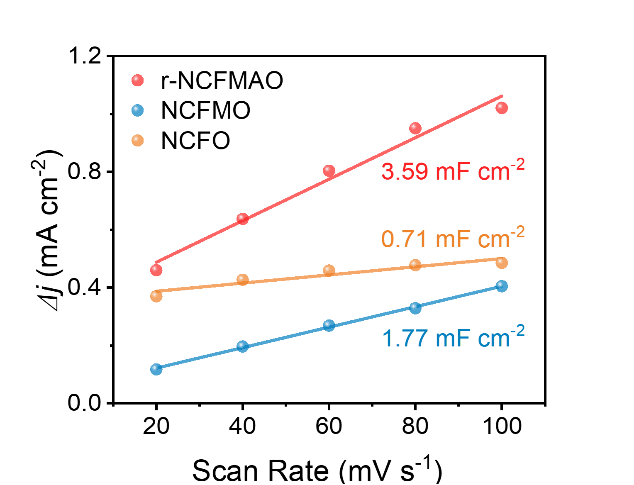


**Figure S15.** The fitting results of ECSA tests of different catalysts in alkaline simulated seawater electrolyte (1.0 M KOH + 0.5 M NaCl + 0.1 M GA).

**
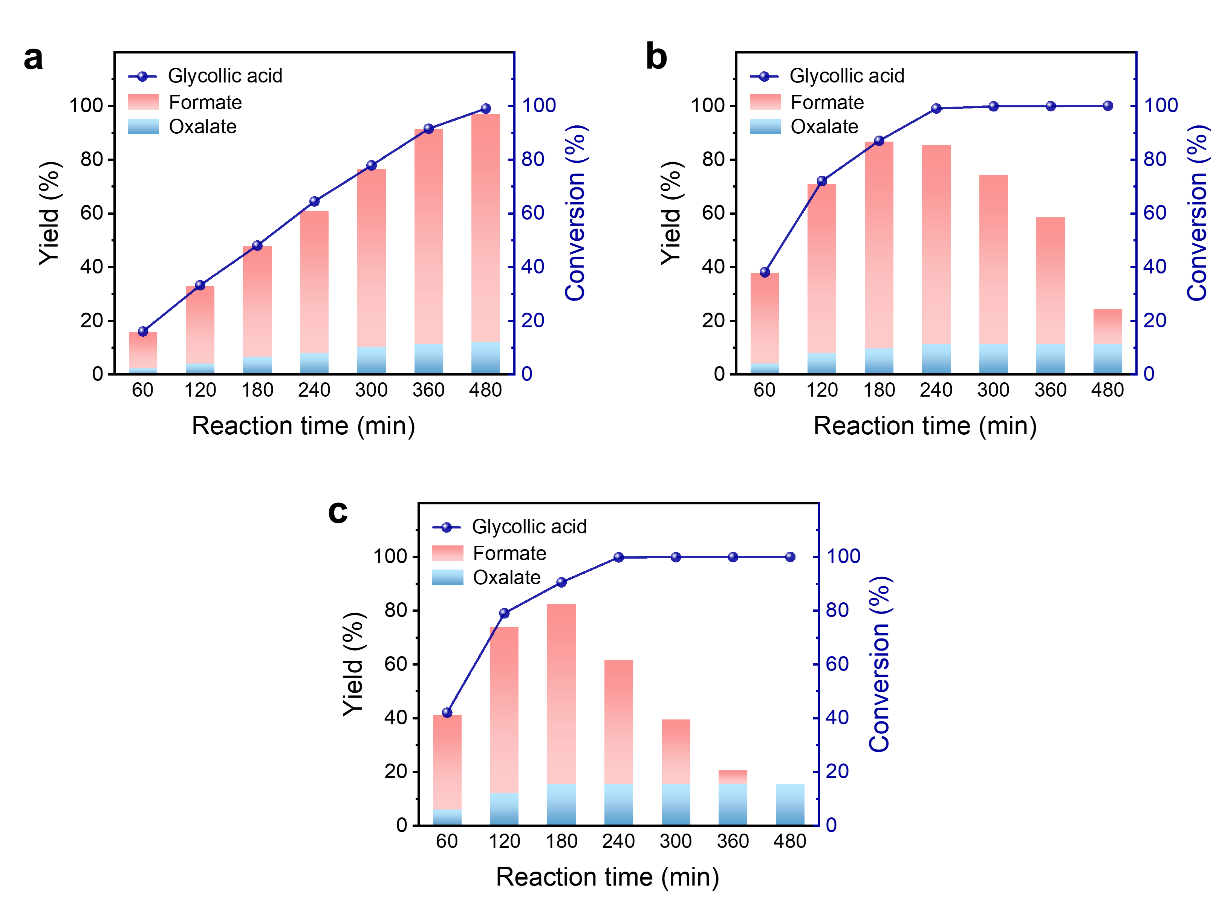
**

**Figure S16.** Conversions of 0.1 M GA and product yields in simulated seawater under constant potential reaction, a) 1.40 V *vs*. RHE, b) 1.45 V *vs*. RHE and c) 1.50 V *vs*. RHE, respectively.

The potentials correspond to data before iR compensation.

**
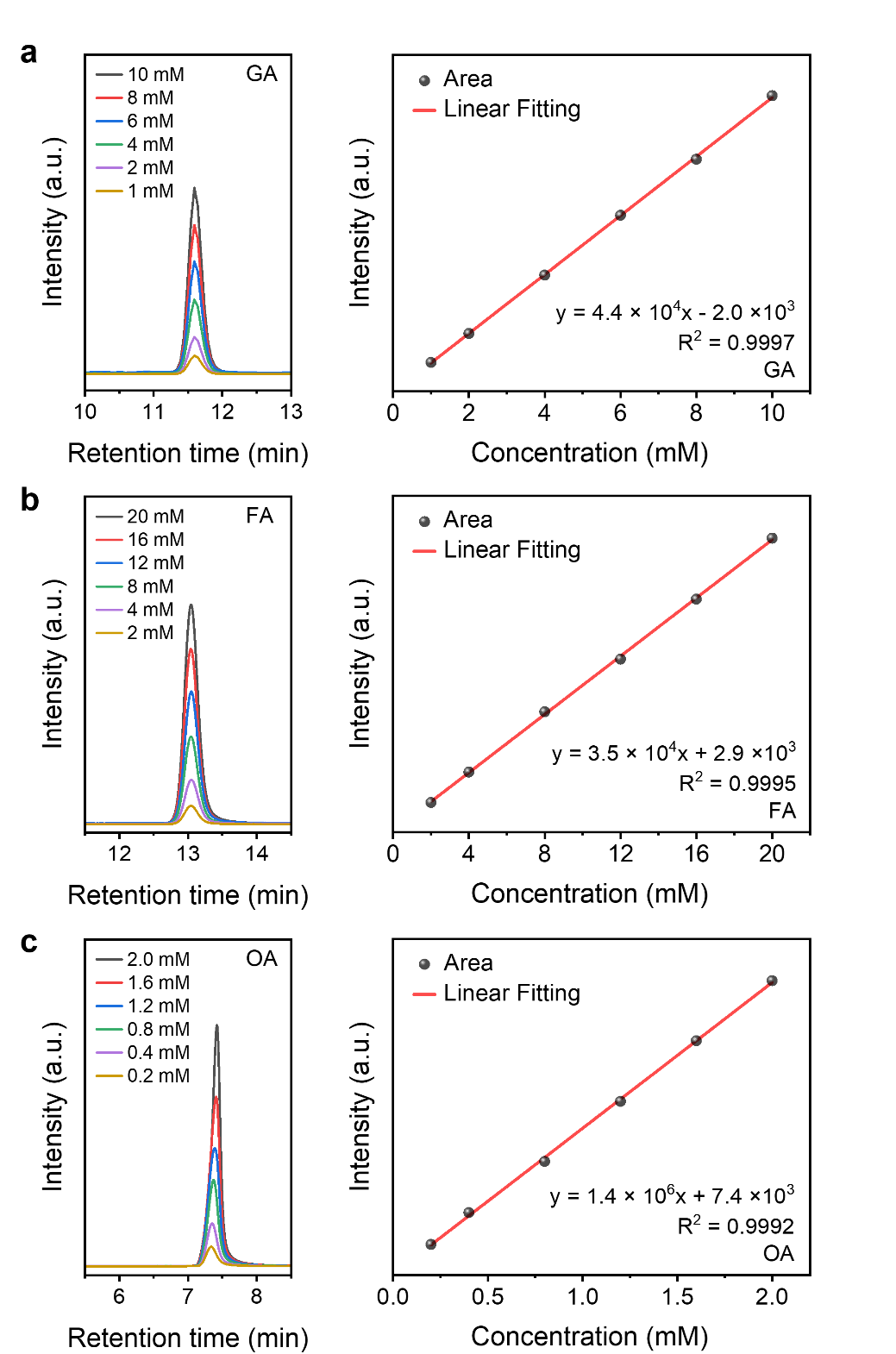
**

**Figure S17.** The HPLC standard curves of a) GA, b) formate and c) oxalate.


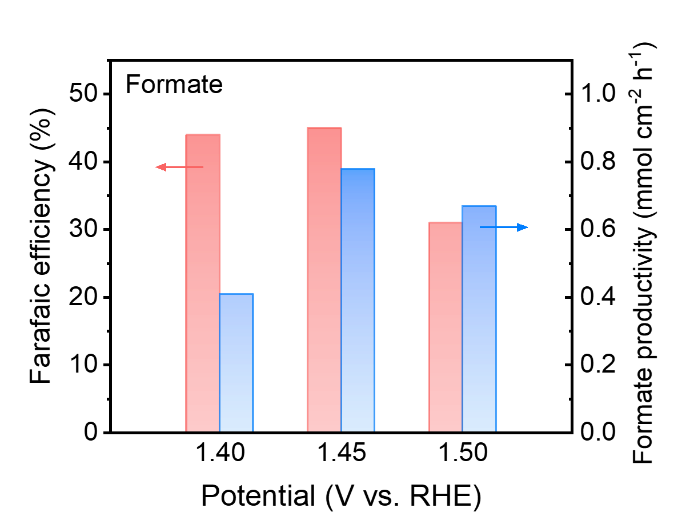


**Figure S18.** Faradaic efficiency and productivity of formate on at different potentials during the first three hours of the reaction.

**
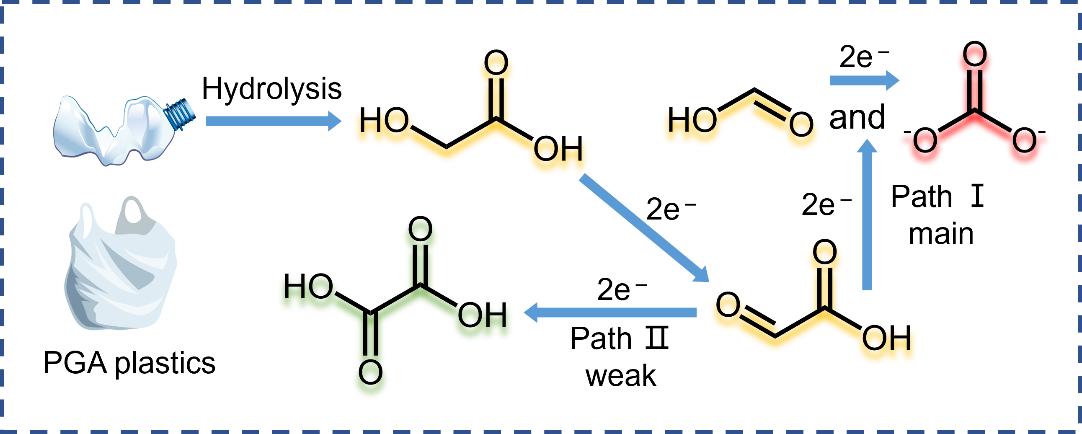
**

**Figure S19.** Hydrolysis and electrocatalytic intermediates and reaction pathways of PGA plastic waste.


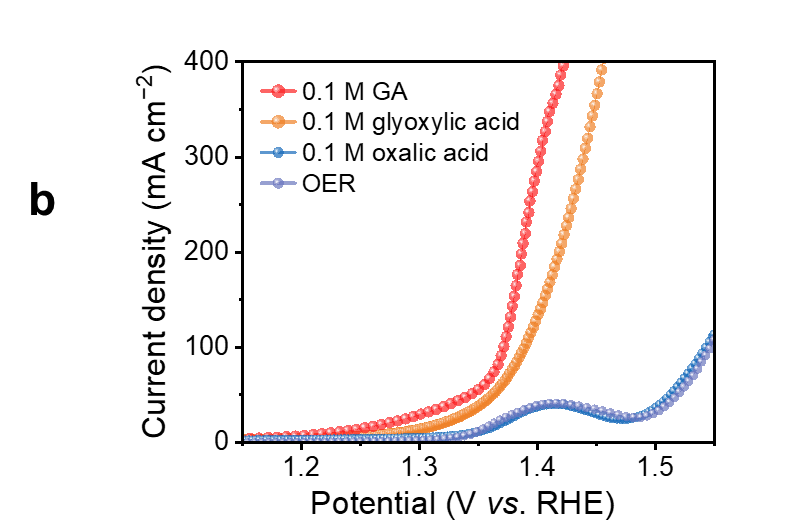


**Figure S20.** LSV curves of glyoxylic acid and oxalate using the r-NCFMAO catalyst in simulated seawater, demonstrating that glyoxylic acid is electrochemically active while oxalate is not, thereby supporting the proposed GAOR pathway.


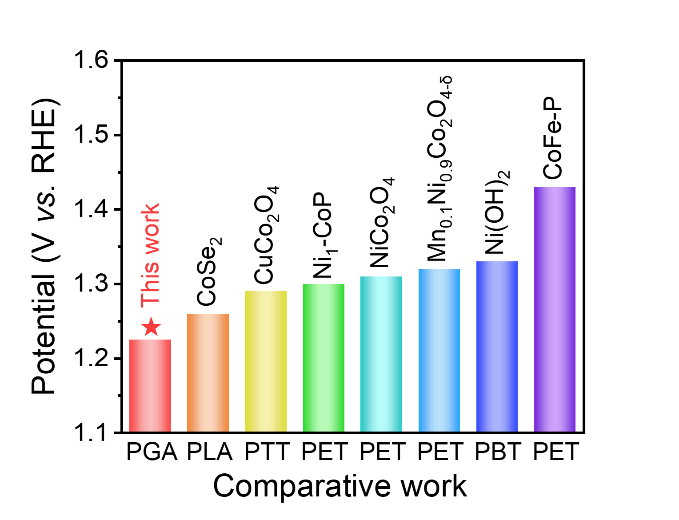


**Figure S21.** The reported non-noble metal catalysts for electrocatalytic treatment of plastics were compared with this work^[4-9]^. The potential corresponded to the current density of 10 mA cm^−2^.


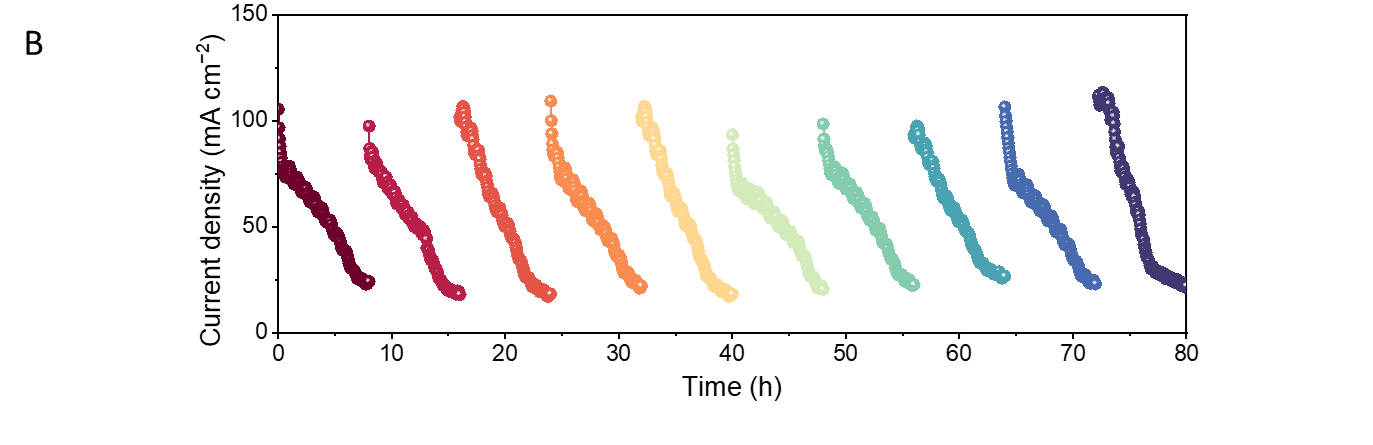


**Figure S22.** Series of i-t curves for the stability test over 10-cycle during which the electrolyte containing PGA hydrolysate was replenished before each cycle.

**
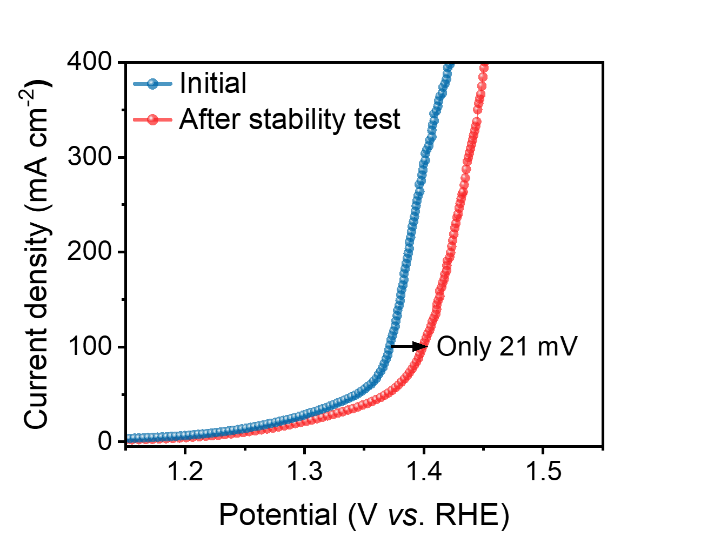
**

**Figure S23.** Comparison of LSV curves for GAOR before and after stabilization of r-NCFMAO.

**
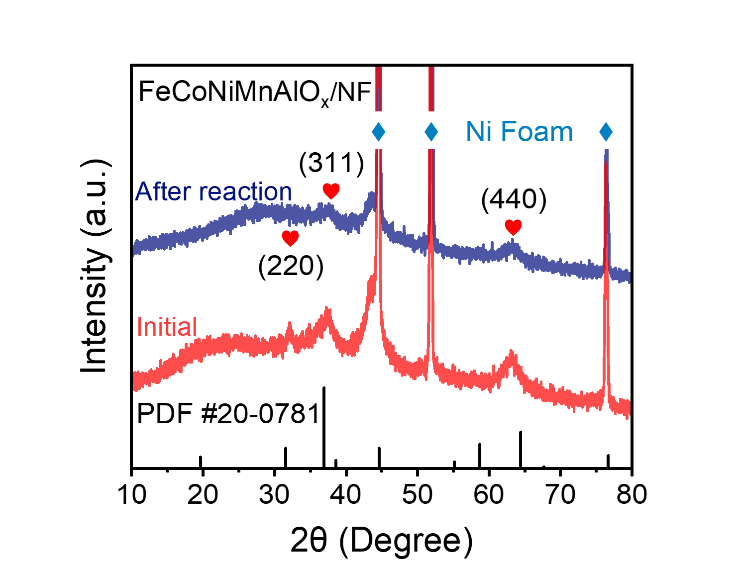
**

**Figure S24.** Comparison of XRD patterns of r-NCFMAO/NF before and after stabilization.

**
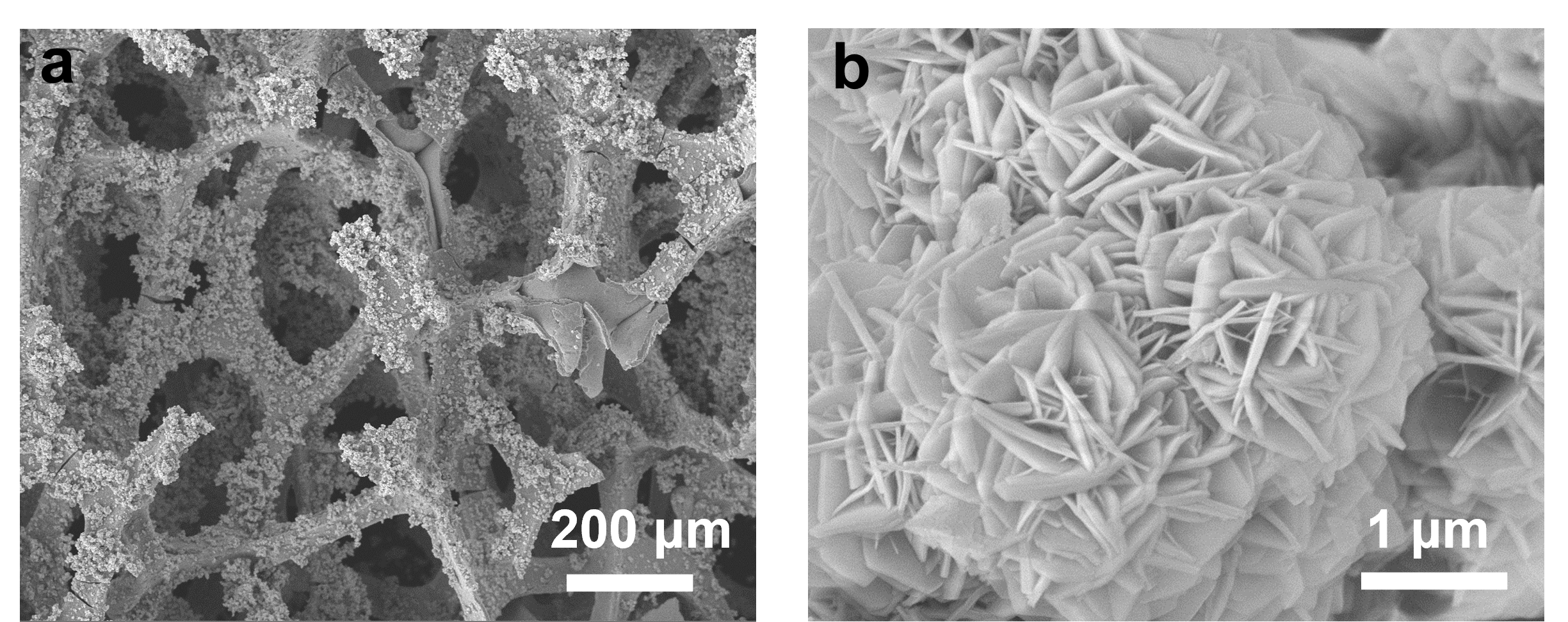
**

**Figure S25.** SEM images of r-NCFMAO after stability test.


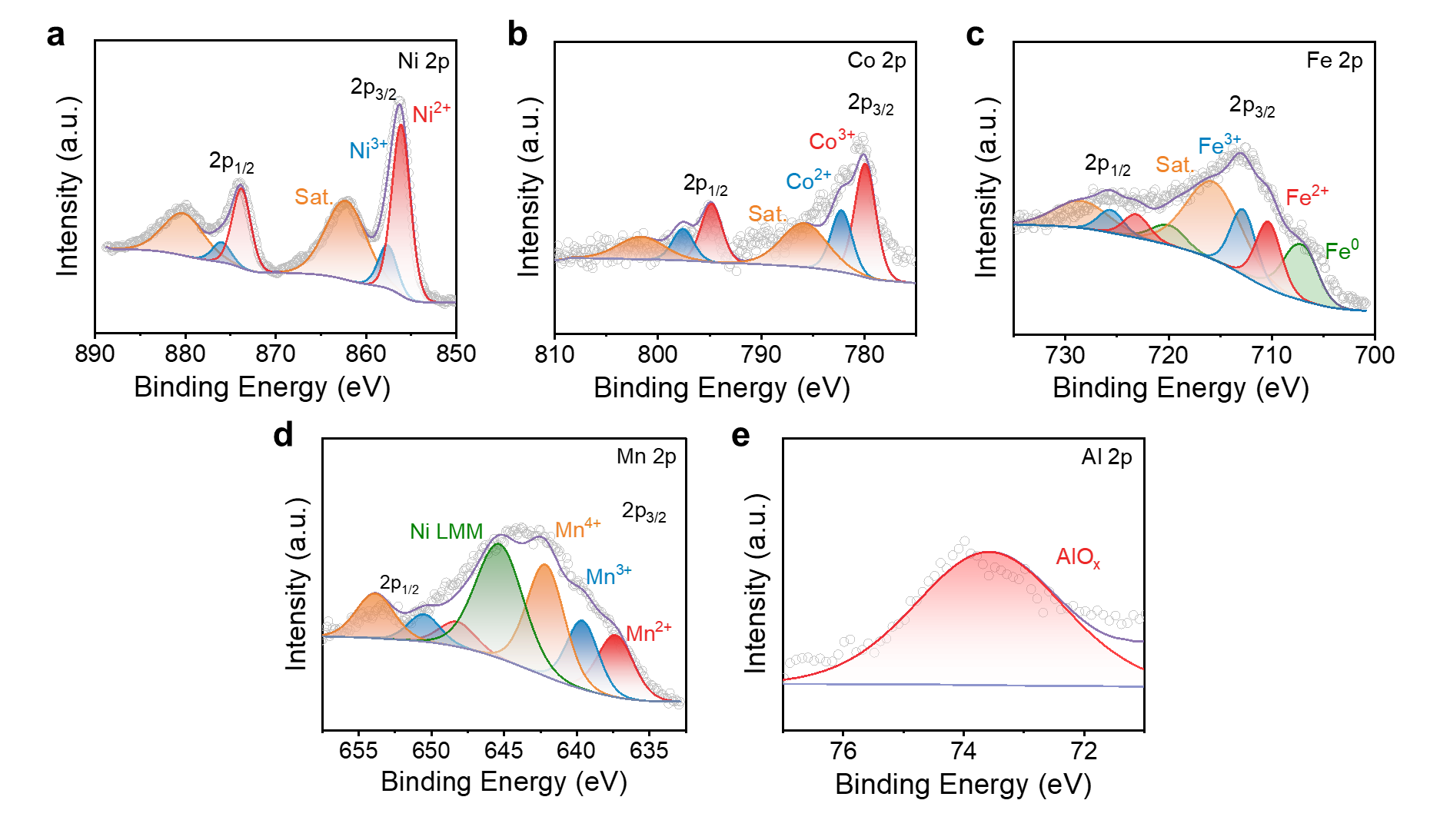


**Figure S26**. XPS spectra of the r-NCFMAO electrode after stability reaction (10 cycles).


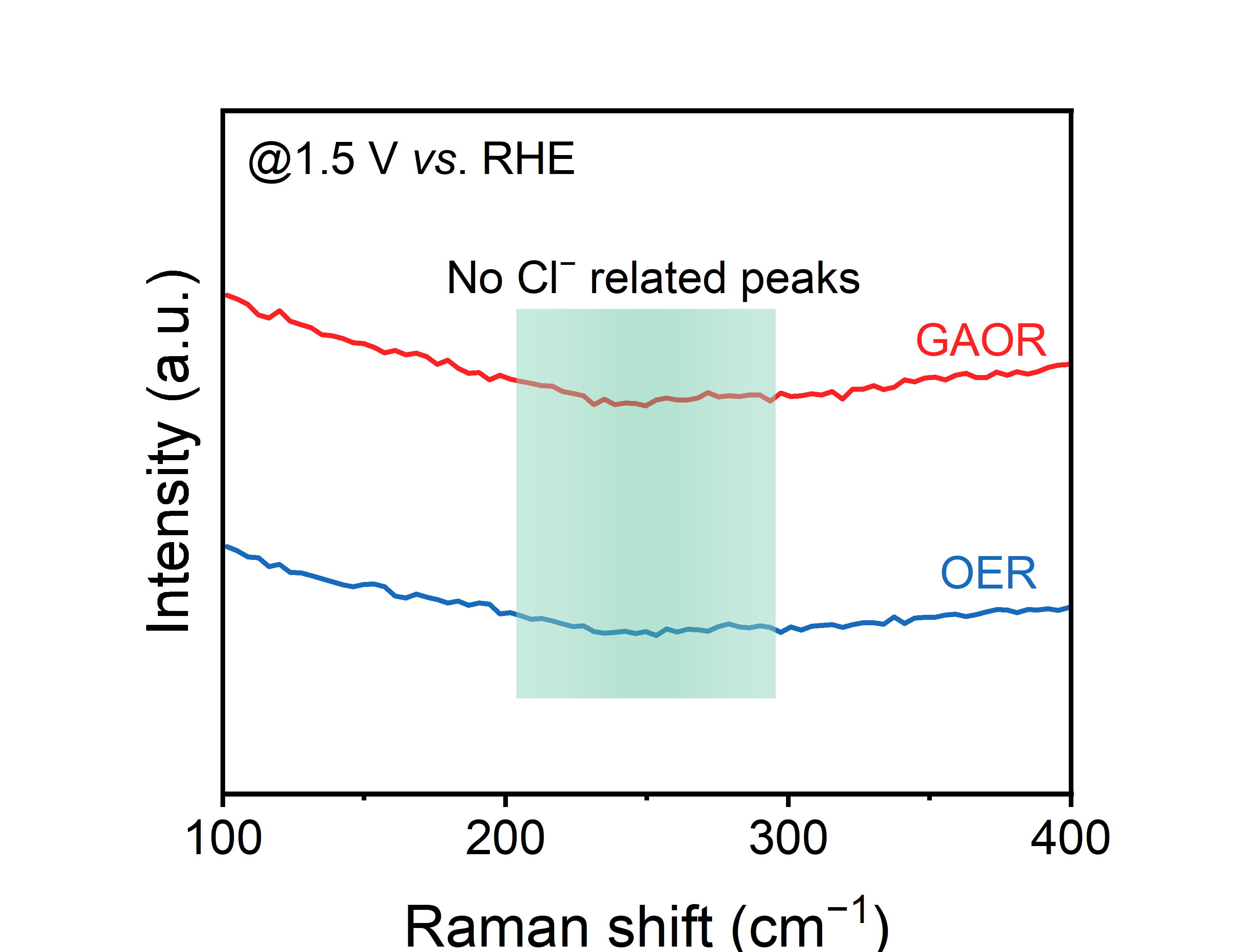


**Figure S27**. *In-situ* Raman spectra of GAOR and OER at 1.5 V *vs*. RHE in simulated seawater.


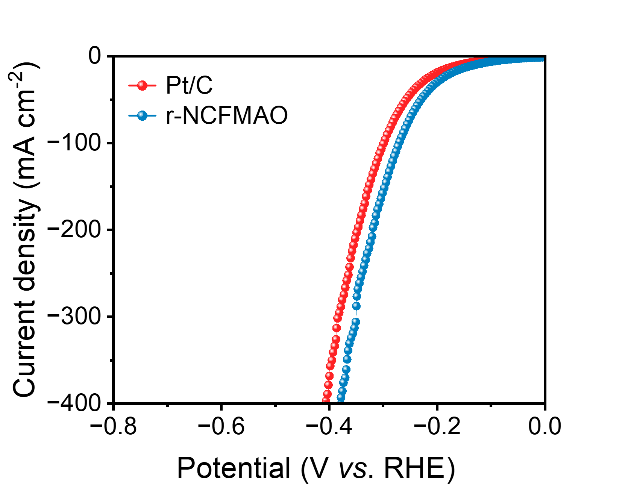


**Figure S28.** Comparison of HER performance between r-NCFMAO and commercial Pt/C catalysts.

**
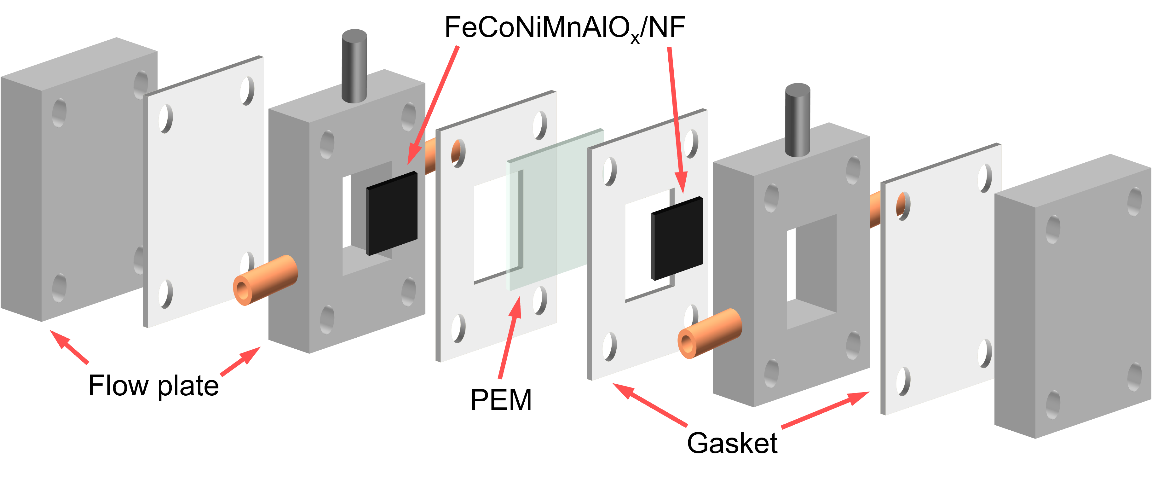
**

**Figure S29.** The assembly diagram of the flow cell pairing system.

**
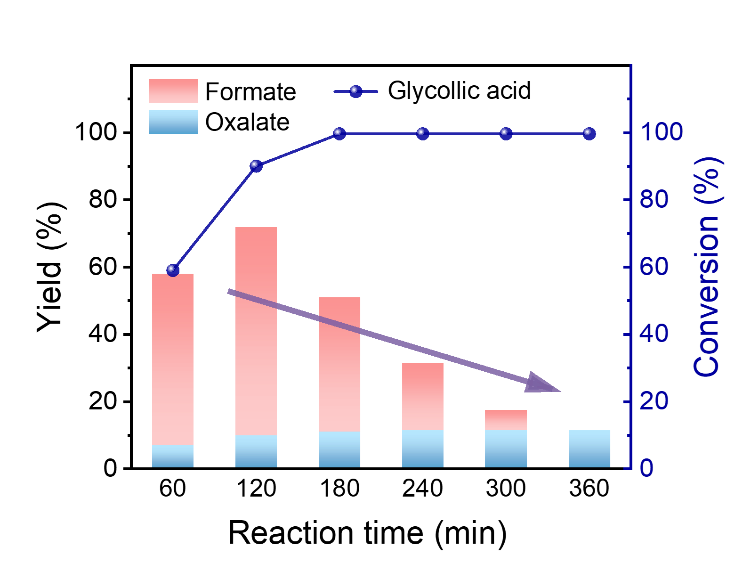
**

**Figure S30.** The HPLC analysis of the anode in two-electrode electrolytic cell for the chronoamperometric test at 1.60 V.


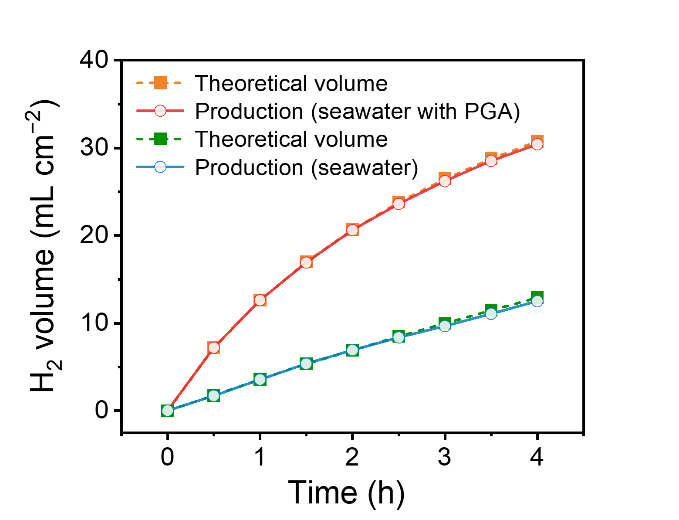


**Figure S31.** In the pairing system, the actual and theoretical hydrogen volume during the reaction process.

**
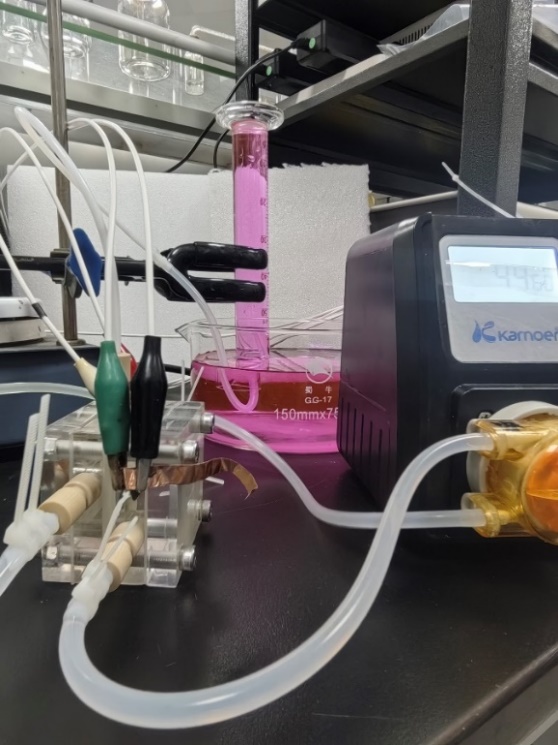
**

**Figure S32.** Diagram of an actual experiment to collect and measure the volume of gas produced at the cathode.


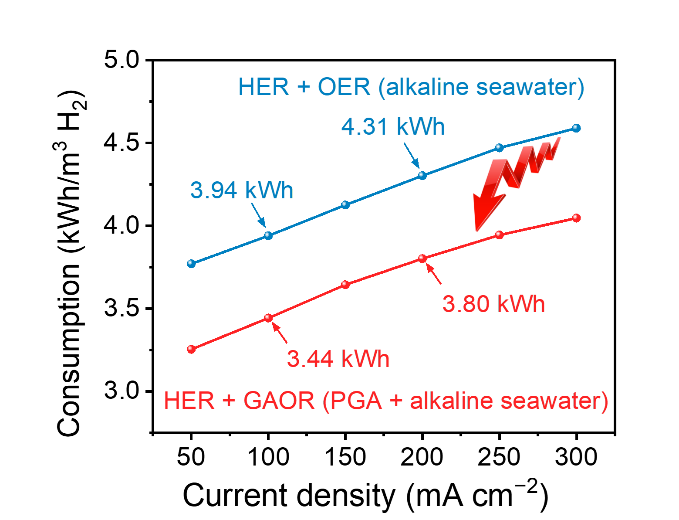


**Figure S33.** The energy consumption of electrocatalytic hydrogen production was calculated by the LSV curve of the paired system^[10]^.


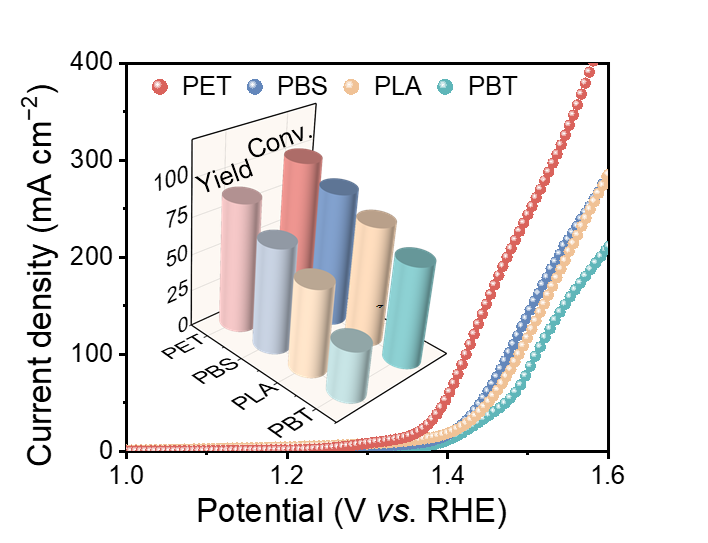


**Figure S34.** The LSV curves of various plastics degraded by r-NCFMAO, including PET, PBS, PLA, and PBT.

**Table S1.** The surface atom compositions of r-NCFMAO from XPS

| Elemental | Ni | Co | Fe | Mn | Al | O |
| --- | --- | --- | --- | --- | --- | --- |
| Composition | 15.7% | 17.7% | 13.9% | 17.6% | 10.5% | 24.4% |

**References**

[1] G. Kresse, D. Joubert, From Ultrasoft Pseudopotentials to the Projector Augmented-Wave Method, 1999, Phys. Rev. B, 59, 1758-1775, 10.1103/PhysRevB.59.1758.

[2] J. P. Perdew, K. Burke, M. Ernzerhof, Generalized Gradient Approximation Made Simple, 1996, Phys. Rev. Lett., 77, 3865-3868, 10.1103/PhysRevLett.77.3865.

[3] S. Grimme, J. Antony, S. Ehrlich, H. Krieg, A Consistent and Accurate Ab Initio Parametrization of Density Functional Dispersion Correction (DFT-D) for the 94 Elements H-Pu, 2010, J. Chem. Phys., 132, 154104, 10.1063/1.3382344.

[4] J. Wang, X. Li, M. Wang, T. Zhang, X. Chai, J. Lu, T. Wang, Y. Zhao, D. Ma, Electrocatalytic Valorization of Poly(Ethylene Terephthalate) Plastic and CO_2_ for Simultaneous Production of Formic Acid, 2022, ACS Catal., 12, 6722-6728, 10.1021/acscatal.2c01128.

[5] F. Liu, X. Gao, R. Shi, E. C. M. Tse, Y. Chen, A General Electrochemical Strategy for Upcycling Polyester Plastics into Added-Value Chemicals by a CuCo_2_O_4_ Catalyst, 2022, Green Chem., 24, 6571-6577, 10.1039/D2GC02049A.

[6] J. Chang, L. Wang, D. Wu, F. Xu, K. Jiang, Y. Guo, Z. Gao, Concurrent Electrocatalytic Hydrogen Evolution and Polyethylene Terephthalate Plastics Reforming by Self-Supported Amorphous Cobalt Iron Phosphide Electrode, 2024, J. Colloid Interface Sci., 655, 555-564, 10.1016/j.jcis.2023.11.044.

[7] X. Liu, J. Wang, Z. Fang, S. Gong, D. Xiong, W. Chen, D. Wu, Z. Chen, Ultrafast Activation of Ni Foam by Electro-Corrosion and Its Use for Upcycling PBT Plastic Waste, 2023, Appl. Catal. B Environ., 334, 122870, 10.1016/j.apcatb.2023.122870.

[8] N. Wang, X. Li, M.-K. Hu, W. Wei, S.-H. Zhou, X.-T. Wu, Q.-L. Zhu, Ordered Macroporous Superstructure of Bifunctional Cobalt Phosphide with Heteroatomic Modification for Paired Hydrogen Production and Polyethylene Terephthalate Plastic Recycling, 2022, Appl. Catal. B Environ., 316, 121667, 10.1016/j.apcatb.2022.121667.

[9] Y. Mao, S. Fan, X. Li, J. Shi, M. Wang, Z. Niu, G. Chen, Trash to Treasure: Electrocatalytic Upcycling of Polyethylene Terephthalate (PET) Microplastic to Value-Added Products by Mn_0.1_Ni_0.9_Co_2_O_4-δ_ RSFs Spinel, 2023, J. Hazard. Mater., 457, 131743, 10.1016/j.jhazmat.2023.131743.

[10] L. Guo, J. Chi, T. Cui, J. Zhu, Y. Xia, H. Guo, J. Lai, L. Wang, Phosphorus Defect Mediated Electron Redistribution to Boost Anion Exchange Membrane-Based Alkaline Seawater Electrolysis, 2024, Adv. Energy Mater., 14, 2400975, 10.1002/aenm.202400975.
